# Distinct proteomic profiles in prefrontal subareas of elderly major depressive disorder and bipolar disorder patients

**DOI:** 10.1038/s41398-022-02040-7

**Published:** 2022-07-11

**Authors:** Yang-Jian Qi, Yun-Rong Lu, Li-Gen Shi, Jeroen A. A. Demmers, Karel Bezstarosti, Erikjan Rijkers, Rawien Balesar, Dick Swaab, Ai-Min Bao

**Affiliations:** 1 NHC and CAMS key laboratory of Medical Neurobiology, School of Brain Science and Brain Medicine, Department of Neurology of the Second Affiliated Hospital, Zhejiang University School of Medicine, Hangzhou, PR China; 2 Department of Psychiatry, Second Affiliated Hospital, School of Medicine, Zhejiang University, Hangzhou, China; 3 Department of Neurosurgery, Second Affiliated Hospital, School of Medicine, Zhejiang University, Hangzhou, China; 4 Proteomics Center, Erasmus University Medical Center, Rotterdam, The Netherlands; 5 Netherlands Institute for Neuroscience, an Institute of the Royal Netherlands Academy of Arts and Sciences, Meibergdreef 47, 1105 BA Amsterdam, The Netherlands

**Keywords:** Molecular neuroscience, Long-term memory

## Abstract

We investigated for the first time the proteomic profiles both in the dorsolateral prefrontal cortex (DLPFC) and anterior cingulate cortex (ACC) of major depressive disorder (MDD) and bipolar disorder (BD) patients. Cryostat sections of DLPFC and ACC of MDD and BD patients with their respective well-matched controls were used for study. Proteins were quantified by tandem mass tag and high-performance liquid chromatography-mass spectrometry system. Gene Ontology terms and functional cluster alteration were analyzed through bioinformatic analysis. Over 3000 proteins were accurately quantified, with more than 100 protein expressions identified as significantly changed in these two brain areas of MDD and BD patients as compared to their respective controls. These include OGDH, SDHA and COX5B in the DLPFC in MDD patients; PFN1, HSP90AA1 and PDCD6IP in the ACC of MDD patients; DBN1, DBNL and MYH9 in the DLPFC in BD patients. Impressively, depending on brain area and distinct diseases, the most notable change we found in the DLPFC of MDD was ‘suppressed energy metabolism’; in the ACC of MDD it was ‘suppressed tissue remodeling and suppressed immune response’; and in the DLPFC of BD it was differentiated ‘suppressed tissue remodeling and suppressed neuronal projection’. In summary, there are distinct proteomic changes in different brain areas of the same mood disorder, and in the same brain area between MDD and BD patients, which strengthens the distinct pathogeneses and thus treatment targets.

## Introduction

Major depressive disorder (MDD) and bipolar disorder (BD) are two major kinds of (mood) disorders [1]. The clinical differences between these syndromes, i.e., depression and mania vs only depression, indicate differences in their respective pathogenesis. In the past, by investigation of the postmortem human brain material, our group and others have observed multiple molecular changes at the mRNA level in the prefrontal regions of MDD and BD patients, e.g., in the dorsolateral prefrontal cortex (DLPFC) and the anterior cingulate cortex (ACC) [2–4]. Indeed, these two brain areas are crucially involved in mood control and are the targets for repetitive transcranial magnetic stimulation (rTMS) [5, 6] or deep brain stimulation in clinics [7–9]. It is clear, however, that transcription changes do not necessarily reflect the changes in protein expression, while single protein changes cannot reflect the protein network interaction changes via which proteins exert their function. A comparative analysis of the proteomes of the same brain areas between MDD and BD could result in better understanding of the distinct neuropathogenesis of the two mood disorders.

At present, systematic understanding of complex cellular and molecular events can be greatly facilitated by comprehensive proteomic analyses [10, 11]. Our proteomic data covered more than 3000 proteins and identified more than 100 significantly different proteins as compared to controls, including OGDH, SDHA and COX5B in the DLPFC of MDD patients; PFN1, HSP90AA1 and PDCD6IP in the ACC of MDD patients; DBN1, DBNL and MYH9 in the DLPFC of BD patients. We observed clear differences between the DLPFC and ACC proteome profiles not only in MDD but also in BD. In addition, clear differences were observed between MDD and BD. Gene ontology (GO) analysis revealed that proteins in different signaling pathways were affected. In the DLPFC of MDD there was significantly decreased energy metabolism, while in ACC were decreased activities of tissue remodeling, immune response and synaptic function. In the DLPFC of BD were decreased activities of tissue remodeling and neuronal projection, while in ACC no specific functional cluster change was observed. To the best of our knowledge, this is the first/most comprehensive methodical comparative proteomic analysis between different brain areas and distinct mood disorders to date. Our results provide unique insights into the protein-related biological pathway changes which are dependent on both brain region and type of mood disorder, laying a solid foundation for future mood disorder research.

## Material and methods

### Brain material and quantification of the percentages of grey matter and white matter

Brain material was obtained from the Netherlands Brain Bank (NBB) using well standardized protocols with written informed consent obtained by the NBB for a brain autopsy and for the use of the material and clinical information for research purposes [12, 13]. The patients had been diagnosed in psychiatric clinics either as MDD or BD during their lifetime according to the DSM-IV criteria, and the diagnosis was confirmed on the basis of the DSM-IV criteria by a board-certified psychiatrist using extensive medical records. The control subjects had not suffered from any primary neurological disorder, other psychiatric disease, or alcohol abuse. The absence of neuropathological changes, both in the MDD or BD patients and in the controls, was confirmed by systematic neuropathological investigation [14]. The MDD and BD patients were respectively well-matched with their respective controls for the following factors: brain area, sex, age, postmortem delay, clock time of death, month of death, cerebrospinal fluid pH and brain weight. In addition, the distribution of patients who died of legal euthanasia was not significantly different among the groups (MDD- DLPFC, 4/16, MDD-CTR- DLPFC, 2/16; BD- DLPFC, 0/5, BD-CTR- DLPFC, 2/5; MDD- ACC, 4/12, MDD-CTR- ACC, 1/12; BD- ACC, 1/7, BD-CTR- ACC, 0/7, Chi-square value = 48.000, *p* = 0.243), or among MDD and MDD’s respective controls (Chi-square value = 12.000, *p* = 0.213), or among BD and BD’s respective controls (Chi-square value = 12.000, *p* = 0.213). The sample sizes of different brain areas of BD and of MDD patients were, however, different; therefore we had 16 DLPFC and 12 ACC from different controls for matching the 16 DLPFC and 12 ACC of MDD patients, and 5 DLPFC and 7 ACC from different controls for matching the 5 DLPFC and 7 ACC of BD patients, respectively (See Supplementary Table 1_·_1–1_·2_).

The method of quantification of the percentages of grey matter and white matter was provided in Supplementary Material and Methods.

### Proteomics: from brain tissue homogenization to Nanoflow LC-MS/MS

In this study, we combined tandem mass tag and high-performance liquid chromatography-mass spectrometry (LC-MS/MS) to characterize the proteome of the DLPFC and ACC of MDD and BD patients. DLPFC or ACC sections were homogenized in 200 μl lysis buffer using sonication in a Bioruptor® Pico (Diagenode) with 10 cycles (30 s on, 30 s off, 4 °C) in 1.5 ml Eppendorf cups. Each sample was then heated for 10 min at 85 °C. Next, protein concentrations were determined using the BCA (bicinchoninic acid) assay according to the manufacturer’s instructions. 100 μg protein in 100 μl lysis buffer per brain sample was reduced with 5 mM DTT at 55 °C for 30 min. After cooling the sample to room temperature, 5 μl of a 200 mM IAA solution was added and the sample was incubated for 15 min at RT in the dark. Protein precipitation was performed as previously described [15] to obtain pellet (for details see Supplementary Material and Methods). Dried pellet was resuspended in 100 μl 50 mM EPPS buffer (pH 8.2), followed by adding 0_·_5 μg Lys-C (enzyme: substrate ratio 1:200) and the sample was incubated for 4 h at 37 °C with shaking. Next, 2.5 μg trypsin (enzyme: substrate ratio 1:40) was added to the sample and the sample was incubated overnight at 37 °C. Peptides were labeled with TMT-11plex reagents (Thermo Fisher Scientific) according to the manufacturer’s protocol. The TMT-labeled peptides were pooled dried in a speedvac and desalted on a 50 mg C18 Sep-Pak cartridge (Waters, cat no: WAT054960). Labeled peptides were eluted with 80% acetonitrile (AcN) and fractionated on an Agilent 1100 HPLC system using a 5 μm particle size 4.6 × 250 mm TSKgel amide-80 column (Tosoh Biosciences, cat no: 0021982). 250 μg of TMT-labeled peptides in 80% AcN were loaded onto the column. Peptides were then eluted using a nonlinear gradient from 80% B (100% AcN) to 100% A (20 mM ammonium formate in water) with a flow rate of 1 ml/min. Twenty-four 4 ml fractions were collected, lyophilized, and pooled into six final fractions. Each fraction was then analyzed by nanoflow LC−MS/MS on an Easy nLC 1200 system (Thermo Fisher Scientific) coupled to an Orbitrap Fusion Lumos Tribrid mass spectrometer (Thermo Fisher Scientific), operating in positive mode and equipped with a nanospray source.

### Statistical and Bioinformatic analysis

Data were analyzed with Proteome Discoverer 2_·_3. The Mascot search algorithm (version 2.6.2, Matrix Science) was used for searching against the UniProt database (taxonomy: Homo sapiens, version April 2019). Following up quantitative analysis, MaxQuant (version 1.5.4.1) data were imported in the Perseus software suite [16]. The statistical analysis was performed in IBM SPSS (version 26). SPSS was used for analysis of grey matter/white matter ratio, and for comparing the expression levels of specific cell type markers, followed by the Bonferroni correction for multiple testing as a post-hoc test. All the tests were two-sided. Spearman Correlation was used to analyze the correlation between the duration of the disorder and the expression levels of the key proteins in different brain areas (DLPFC or ACC) of MDD or BD patients. GO enrichment analysis and Perseus software were used for bioinformatic analysis. Uniprot database was used for searching specific proteins and their functions, and Rstudio (v 3.3.0) was used to calculate the Z-score of specific GO terms. String database was used to analyze the protein network. In addition, CTD database (http://ctdbase.org/) was used to analyze the medication influence on the key proteins. Detailed information of the Mascot search algorithm setting and the type of test used is provided in Supplementary Material and Methods.

### Role of funding source

The funders have no role in study design, data collection, data analysis, interpretation, or writing of the report.

## Results

The ratio of the grey matter/white matter of our samples was around 2.7, indicating the size of grey matter is significantly larger than the white matter (Supplementary Fig. 1A, B). In addition, we found that the neuron signature marker, MAP2, was significantly higher than those of the prototypic markers for astrocytes, oligodendrocytes, endotheliocytes, and macrophages (Supplementary Fig. 2A, B). It should be noted that the cellular proportion (Supplementary Fig. 2) does not need to reflect the involvement of cell types in the disease state, but rather showed the cell-specific markers detected in this proteomics experiment. These findings propose that neurons, rather than glial cells, are the main cell type in our detected samples.

### Distinct functional cluster and protein expression alterations in the DLPFC and ACC of MDD patients compared with their respective controls

**Fig. 1:**
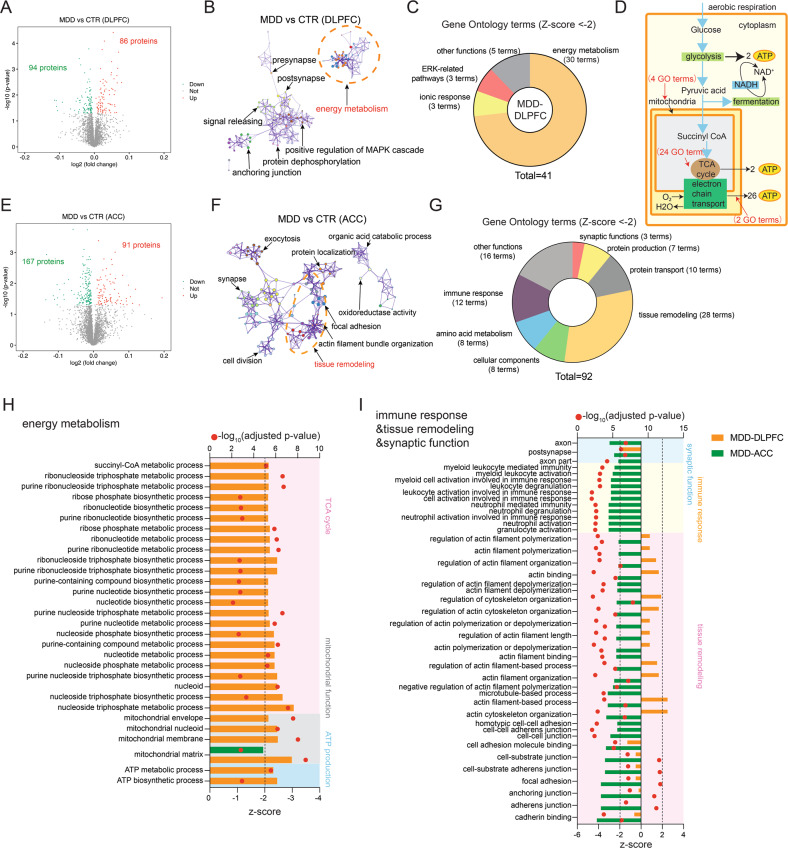
Distinct functional cluster alterations in the dorsolateral prefrontal cortex (DLPFC) and anterior cingulate cortex (ACC) of major depressive disorder (MDD) patients compared with their respective controls. **A** Differential expression analysis was performed on proteomics data. Volcano plot shows the differentially expressed proteins (DEPs; adjusted *p* value < 0_·_05) in the DLPFC of MDD patients compared with controls. 180 DEPs in the DLPFC were identified, among which 94 proteins were downregulated and 86 proteins were upregulated. **B** Gene ontology (GO) enrichment analysis was performed using Metascape on the 140 DEPs in the DLPFC of MDD patients. The significantly overrepresented (adjusted *p* value < 0_·_01) GO terms were grouped into color-coded clusters based on their membership similarities and rendered as a network plot. Each node represents an enriched term, and one representative term is shown for each cluster. Terms with a similarity >0.3 are connected by edges. **C** The numbers of significantly overrepresented GO terms in the DLPFC of MDD patients. Four major functional clusters were identified from 41 GO terms with a z-score < −2. **D** Procedure of aerobic respiration. The number of GO terms that are related to specific steps are indicated. **E** Volcano plot shows the DEPs in the ACC of MDD patients compared with controls. 258 DEPs in the DLPFC were identified, among which 167 proteins were downregulated and 91 proteins were upregulated. **F** GO enrichment analysis shows the most involved functional cluster is tissue remodeling in the ACC of MDD patients. **G** The numbers of significantly overrepresented GO terms in the ACC of MDD patients. Seven major functional clusters were identified from 92 GO terms with a z-score < −2. **H** The suppression status of biological processes related to energy metabolism was assessed by calculating their suppression z-scores using GOplot. The 30 GO terms are shown associated with energy metabolism that were predicted to be strongly decreased (z-score < −2) in the DLPFC of MDD patients. None of them were predIcted to be strongly decreased (z-score < −2) in the ACC of MDD patients. **I** The suppression status of biological processes related to immune responses, tissue remodeling and synaptic function was assessed by calculating their suppression z-scores using GOplot. The 39 GO terms associated with immune responses, cytoskeleton organization, adhesion process and synaptic function were predicted to be strongly decreased (z-score < −2) in the ACC of MDD patients. None of them were predicted to be strongly decreased (z-score < -2) and two of them were predicted to be strongly activated (z-score >2) in the DLPFC of MDD patients.

To address the proteomic variation in the DLPFC and ACC of MDD patients, the abundance of each protein of patients was compared with their respective DLPFC and ACC of controls. Compared with their respective controls, we found more differentially expressed proteins (DEPs) in the ACC (258) than in the DLPFC (180) (Fig. 1A, E).

With the intention of identifying the most altered pathways, we performed GO analysis and observed that in the DLPFC, a total of 41 GO terms were significantly overrepresented by the DEPs (Supplementary Table 2), with the most enriched functional cluster being energy metabolism. Among the 30 GO terms related to energy metabolism, two were related to ATP production, 24 to the tricarboxylic acid (TCA) cycle and four to mitochondrial function (Fig. 1D). Compared with energy metabolism, other functional cluster changes were not evident, i.e., containing much less GO terms (Fig. 1B, C). Striking differences were found in the ACC compared with the DLPFC: a total of 92 GO terms were significantly overrepresented (Supplementary Table 4), with the most enriched functional cluster being tissue remodeling, followed by immune response (Fig. 1F, G). Also, 3 GO terms related to synaptic function were found to be suppressed in the ACC of MDD patients. There was no overlap of suppressed GO terms in relation to energy metabolism, tissue remodeling or immune response between the DLPFC and ACC (Fig. 1H, I).

**Fig. 2:**
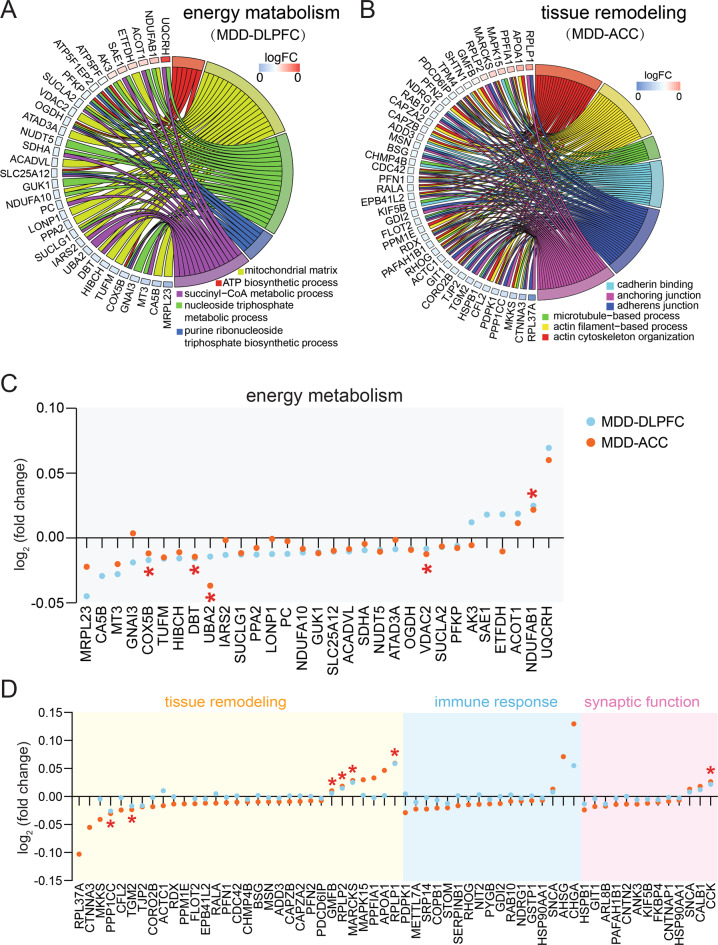
Different protein expressions were altered in the dorsolateral prefrontal cortex (DLPFC) and anterior cingulate cortex (ACC) of major depressive disorder (MDD) patients compared with their respective controls. **A** Proteins related to energy metabolism were explored for their involvement in five functional sub-categories. Shown are proteins associated with at least two sub-categories, displayed as a Circos plot. **B** Proteins related to tissue remodeling were explored for their involvement in 6 functional sub-categories. Shown are proteins associated with at least two sub-categories, displayed as a Circos plot. **C** Expression profiles of significantly changed energy metabolism-related proteins in the DLPFC of MDD patients. Only five of them were significantly changed in the ACC of MDD patients. **D** Expression profiles of significantly changed immune response, tissue remodeling and synaptic function-related proteins in the ACC of MDD patients. Only seven of them were significantly changed in the DLPFC of MDD patients.

We further subjected the protein expression changes to GOplot with the purpose to elucidate the relationship between specific proteins and biological processes. We observed multiple downregulated key proteins of energy metabolism in the DLPFC of MDD, including PFKP, OGDH, SDHA, NDUFA10 and COX5B, which were clustered in at least two energy metabolism functions and were also hub proteins in the protein network (Fig. 2A, Supplementary Fig. 3). However, in the ACC of MDD there were multiple downregulated key proteins of tissue remodeling, including MSN, PFN1, and PDCD6IP, clustered in at least 3 tissue remodeling functions and were hub proteins in the protein network (Fig. 2B, Supplementary Fig. 4). In addition, several key proteins related to immune response were found to be downregulated, including METTL7A, SRP14 and COPB1, which were clustered in all the immune response functions (Fig. 2D). More energy metabolism-related proteins showing significant expression changes were found in the DLPFC of MDD than in the ACC of MDD (Fig. 2C), while more tissue remodeling-related, immune response-related or synaptic function-related proteins showing significant expression changes were found in the ACC of MDD than in the DLPFC of MDD (Fig. 2D). It should be noted that no significant correlations were found between the duration of the MDD and the expression levels of these key proteins in the DLPFC (p≧0.220) or ACC (p≧0.155).

Taken together, these results indicated distinct proteomics alteration in the DLPFC and the ACC of MDD patients.

### Distinct DLPFC functional cluster and protein expression alterations in MDD and BD patients compared with their respective controls

**Fig. 3:**
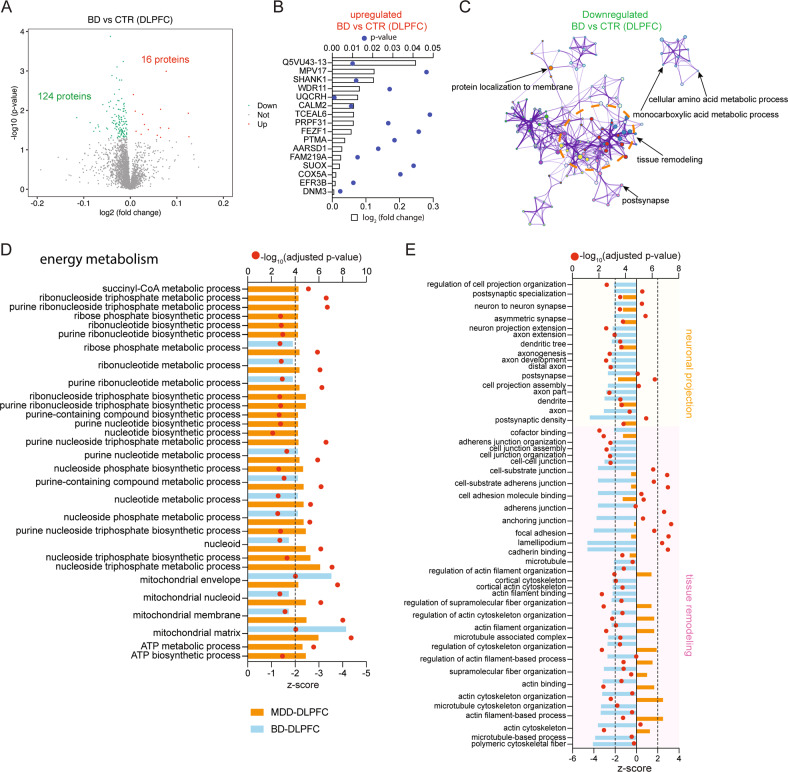
Distinct dorsolateral prefrontal cortex (DLPFC) functional cluster alterations in major depressive disorder (MDD) and bipolar disorder (BD) patients compared with their respective controls. **A** Volcano plot shows the differentially expressed proteins (DEPs; adjusted *p* value < 0_·_05) in the DLPFC of BD patients compared with controls. 140 DEPs in the DLPFC were identified, among which 124 proteins were downregulated and 16 proteins were upregulated. **B** Expression profiles of the 16 upregulated DEPs in BD patients compared with controls. **C** Gene Ontology (GO) enrichment analysis shows the most involved functional cluster is tissue remodeling in the DLPFC of BD patients. **D** The suppression status of biological processes related to energy metabolism was assessed by calculating their suppression z-scores using GOplot. The biological processes associated with energy metabolism are indicated that were predicted to be strongly decreased (z-score < −2) in the DLPFC of MDD patients. Only six of them were predicted to be strongly decreased (z-score < −2) in the DLPFC of BD patients. **E** The suppression status of biological processes related to neuronal projection and tissue remodeling was assessed by calculating their suppression z-scores using GOplot. The biological processes associated with neuronal projection and tissue remodeling that were predicted to be strongly decreased (z-score < −2) in the DLPFC of BD patients. None of them were predicted to be strongly decreased (z-score < −2) and only two of them were predicted to be strongly activated (z-score >2) in the DLPFC of MDD patients.

140 DEPs were identified in the DLPFC of BD (Fig. 3A), which was fewer than the 180 DEPs identified in the DLPFC of MDD (Fig. 1A). It should be noted that only 16 DEPs were found to be upregulated, among which Q5VU43-13, SHANK1 and DNM3 are related to tissue remodeling (Fig. 3B). A total of 123 GO terms were significantly overrepresented by the downregulated DEPs (Supplementary Table 5), with the most enriched functional cluster as tissue remodeling (Fig. 3C), followed by neuronal projection (Supplementary Table 5). The most suppressed 48 GO terms related to tissue remodeling and neuronal projection (Fig. 3E) were not shown as suppressed, while two were even predicted to be activated, in the DLPFC of MDD patients. In addition, six GO terms related to energy metabolism showed suppression in the DLPFC of BD patients (Fig. 3D), in contrast to the 30 suppressed GO terms related to energy metabolism found in the DLPFC of MDD.

**Fig. 4:**
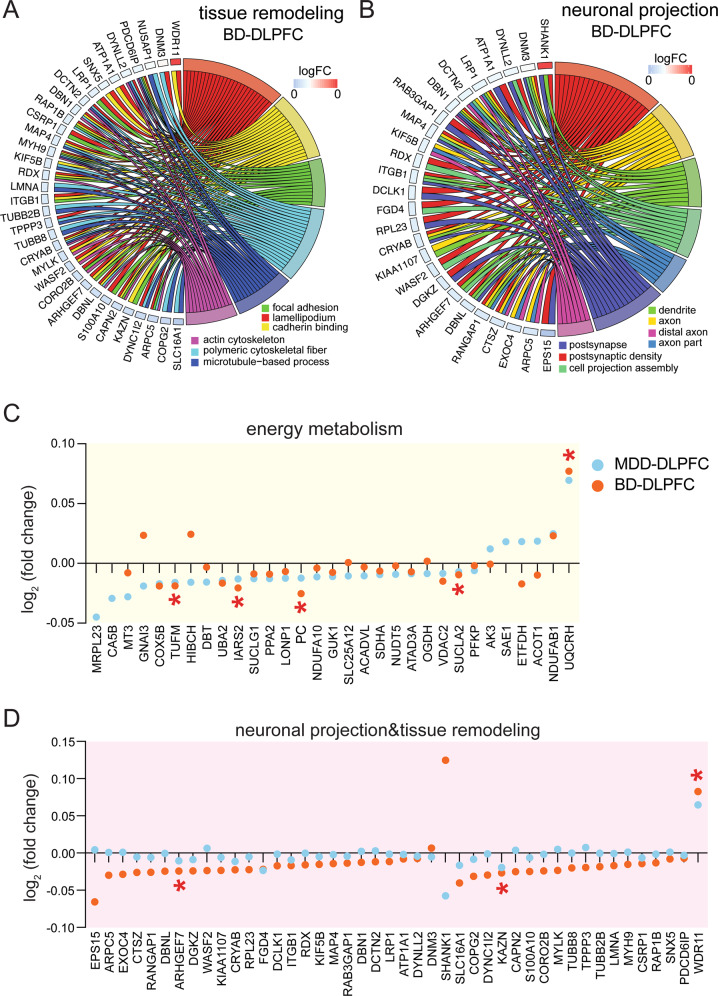
Different protein expressions were altered in the dorsolateral prefrontal cortex (DLPFC) of major depressive disorder (MDD) and bipolar disorder (BD) patients compared with their respective controls. **A** Proteins related to tissue remodeling were explored for their involvement in six functional sub-categories. Shown are proteins associated with at least two sub-categories, displayed as a Circos plot. **B** Proteins related to neuronal projection were explored for their involvement in seven functional sub-categories. Shown are proteins associated with at least two sub-categories, displayed as a Circos plot. **C** Expression profiles of significantly changed energy metabolism-related proteins in the DLPFC of MDD patients. Only five of them were significantly changed in the DLPFC of BD patients. **D** Expression profiles of significantly changed tissue remodeling and neuronal projection-related proteins in the DLPFC of BD patients. Only three of them were significantly changed in the DLPFC of MDD patients.

In the DLPFC of BD, subjecting the protein expression changes to GOplot we observed multiple downregulated key proteins including, MYH9, PDCD6IP, DBN1 and DBNL (Fig. 4A), which were clustered in at least two out of six GO terms of tissue remodeling, and downregulated key proteins including DBN1, ITGB1, ARHGEF7 and DBNL, which were clustered in at least two out of 7 GO terms of neuronal projection, and were hub proteins in the protein network (Fig. 4B, Supplementary Fig. 5). More energy metabolism-related proteins showing significant expression changes were found in the DLPFC of MDD than in the DLPFC of BD (Fig. 4C), while more tissue remodeling-related or neuronal projection-related proteins showing significant expression changes were found in the DLPFC of BD than in the DLPFC of MDD (Fig. 4D). It is also of interest to note that, although the tissue remodeling functional clusters were found to be both suppressed in both the ACC of MDD and the DLPFC of BD, the significantly changed proteins involved were different, except for an overlap of only four proteins, i.e., CORO2B, KIF5B, PDCD6IP and RDX. Taken together, these results indicated distinct proteomics alteration in the DLPFC of MDD and BD patients. It should be noted that no significant correlations were found between the duration of the BD and the expression levels of these key proteins in the DLPFC (*p* ≧ 0.391).

### No significant functional cluster changes found in the ACC of BD patients compared with their respective controls

**Fig. 5:**
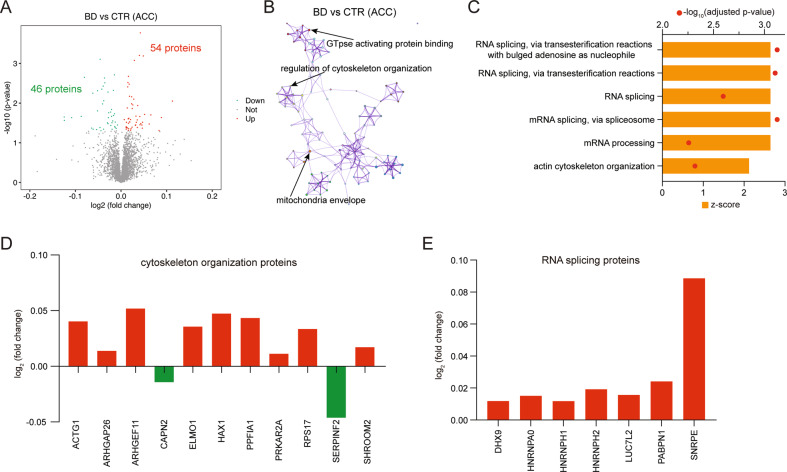
No significant proteomic alterations in the anterior cingulate cortex (ACC) of bipolar disorder (BD) patients compared with their respective controls. **A** Volcano plot shows the differentially expressed proteins (DEPs; adjusted *p* value < 0.05) in the ACC of BD patients compared with controls. **B** Gene Ontology (GO) enrichment analysis shows no specific function cluster is changed in the ACC of BD patients. **C** The activation status of biological processes was assessed by calculating their activation z-scores using GOplot. Shown are the biological processes that were predicted to be strongly activated (z-score >2). **D**, **E** Expression profiles of representative cytoskeleton organization proteins and RNA splicing proteins in the ACC of BD patients. Please note that, in panel (**D**), only two of the cytoskeleton organization proteins were downregulated, while nine were upregulated. In panel (**E**), all seven synapse-related proteins were upregulated.

Although 100 DEPs were identified (Fig. 5A), only 6 GO terms were significantly overrepresented by the DEPs (Supplementary Table 6) and were predicted to be activated (z-score >2) (Fig. 5B, C). No specific functional cluster was found to show significant change in the ACC of BD. The significantly changed proteins related to cytoskeleton organization and RNA splicing are shown in Fig. 5D, E.

The medication influence on the key proteins analyzed by the CTD (http://ctdbase.org/) database showed that clozapine may lead to increased expression of PFKP; haloperidol may lead to increased expression of PFKP and PFN1 (Supplementary Table 7).

### Direct comparison between BD and MDD patients confirmed the findings of their respective comparison with their respective controls

As we mentioned above, since the numbers of samples differed between MDD and BD patients, i.e. MDD DLPFC (*n* = 16) vs BD DLPFC (*n* = 5), and MDD ACC (*n* = 12) vs BD ACC (*n* = 7), we compared these samples with their respective controls of the same sample sizes, respectively. We also checked the direct comparison between MDD and BD in terms of ontology, and we observed that when MDD DLPFC (*n* = 16) was compared with BD DLPFC (*n* = 5), there were 105 DEPs, while when MDD ACC (*n* = 12) was compared with BD ACC (*n* = 7), there were 177 DEPs (Supplementary Fig. 6A, E). Further GO analysis and Z-scores of specific GO terms calculated by R package indicated a relatively activated synaptic function in the DLPFC of MDD compared with the DLPFC of BD, while a relatively lower vesicle function was found in the ACC of MDD compared with the ACC of BD patients (details see Supplementary Material, Supplementary Fig. 6 and Supplementary Table 8). These data were in agreement with the results found when MDD and BD were compared with their respective controls.

## Discussion

It has long been proposed that MDD and BD have distinct biological mechanisms because they show different clinical symptoms. This proposal needed, however, to be verified, especially by studies on postmortem human brains of these mood disorder patients. In this study, for the first time, we systematically investigated the proteomes of DLPFC and ACC of MDD and BD patients with their *respective* well-matched controls. We showed differences in protein profiling depending on brain area and subtype of the mood disorder. The most notable change we found in the DLPFC of MDD was suppressed energy metabolism. In the ACC of MDD we found suppressed tissue remodeling and suppressed synaptic function. The most notable changes in the DLPFC of BD were differentiated suppressed tissue remodeling and suppressed neuronal projections.

### Distinct functional cluster and protein expression alterations in the DLPFC and ACC of MDD patients compared with respective controls

In the central executive system, DLPFC is a key hub involved in cognitive control, working memory, and emotion regulation [17, 18]. The significantly decreased energy metabolism we observed in the DLPFC of MDD was in agreement with the clinical symptoms of energy loss, diminished interest or pleasure in almost all activities and decreased appetite in depression [19], supporting the idea that DLPFC plays a crucial role in the symptomatology of MDD [20]. This idea is further supported by fMRI data which indicate that DLPFC activity was decreased and the functional connection between the DLPFC and the amygdala was reduced in untreated MDD patients performing cognitive function tests [21], and that in depressed patients there was widespread reduction in functional connectivity between the left DLPFC and other brain areas [22]. Indeed, MDD patients had reduced ATP levels in the basal ganglia [23, 24] and frontal lobe [25] reflected in 31 ^P^ nuclear magnetic resonance spectroscopy. In addition, rTMS of the DLPFC shows a decrease in depressive severity and an increase in remission rate, even in therapy resistant MDD patients [5, 6]. Impaired mitochondrial function was found to result in decreased electron transport chain (ETC) and ATP production, impaired bioenergetics, apoptosis and oxidative stress [26], while decreased ATP production and decreased ETC enzyme were indeed observed in MDD patients in muscle biopsy tests [27, 28]. Moreover, in cell and animal models of depression, suppressed energy metabolism-related proteins and pathways were observed [29].

The specifically decreased energy metabolism cluster in the DLPFC of MDD included many biological processes such as ATP metabolic process, succinyl-CoA metabolic process, and mitochondrial matrix. Functions of the significantly downregulated key proteins related to the energy metabolism cluster, such as PFKP, OGDH, SDHA, NDUFA10 and COX5B are listed in Supplementary Table 7, among which COX5B and OGDH are involved in most sub-categories of the energy metabolism functional cluster, and only COX5B was also found downregulated in the ACC of MDD It should be noted that all these proteins are involved in oxidative phosphorylation, which is the main step in ATP production, indicating a key molecular mechanism for the decreased energy metabolism in the DLPFC of MDD that warrants further investigation.

Unlike the proteomic profiles with a major suppressed energy metabolism in the DLPFC, the most notable proteomic change in the ACC of MDD was suppressed tissue remodeling, including actin filament polymerization, microtubule-based process and actin cytoskeleton organization, and significant suppressed immune response. Functions of a number of downregulated key proteins related to tissue remodeling in the ACC of MDD, including MSN, PFN1, HSP90AA1, and PDCD6IP are listed in Supplementary Table 7, none of which was found downregulated in the DLPFC of MDD. ACC is crucial not only for cognitive regulation including decision making, inhibition control, and empathy [30–32], but also for emotion regulation related to rewards and punishment [33–35]. Suppressed or disordered synaptic functions caused by cytoskeleton disorders certainly contribute to ACC dysfunction. Cytoskeletal abnormalities were found to cause dendritic regression and dendritic spine decrease which leads to decreased synaptic connectivity in both depressive patients and mice [36–38]. A proteomics study in an animal model of depression reported proteomic alteration in tubulin and actin in the hippocampus and prefrontal cortex [39].

We also observed a number of suppressed biological processes related to immune response in the ACC, but not in the DLPFC, of MDD patients, including myeloid leukocyte mediated immunity, neutrophil degranulation and granulocyte activation. The functions of key proteins related to immune response which were downregulated, including GDI2, COPB1, STOM, HSP90AA1 and PDCD6IP, are listed in Supplementary Table 7. None of them was found to be significantly changed in the DLPFC of MDD patients. It shall be noted that HSP90AA1 participates both in the immune response and synaptic functions. Mood disorders were found to be associated with peripheral immune system alterations with subsequent over-activation of pro-inflammatory cytokines [40, 41]. Indeed, some anti-inflammatory medicines showed antidepressant effects in MDD patients [42–45]. It should be noted that such activation of certain inflammatory responses and the inhibited immune response we observed in the MDD ACC indicate an abnormal, in general inhibited, immune system in mood disorders, which may be the result of an abnormal, often hyper-active neuroendocrine system [46].

Taken together, these results clearly showed the difference between the DLPFC and ACC of MDD patients, which appeals for future elucidation of the different roles of these two brain regions in the pathogenesis, especially in the different symptoms of MDD.

### Distinct DLPFC functional cluster and protein expression alterations in MDD and BD patients compared with their respective controls

Unlike the proteomic profiling change in the DLPFC of MDD, the most notable change in the DLPFC of BD patients was suppressed tissue remodeling, including cadherin binding, actin cytoskeleton organization and actin filament binding. This showed similarity to the suppressed tissue remodeling functional cluster changes in the ACC of MDD. However, there were only 4 proteins overlap, i.e. RDX, CORO2B, KIF5B and PDCD6IP, which reveals clear differences. The downregulated key proteins related to tissue remodeling found in the DLPFC of BD patients included DBN1, MYH9, DBNL, RDX and PDCD6IP. It is also of interest to note that PDCD6IP contributes to the processes of immune response, and also to tissue remodeling in both the ACC of MDD (see above) and the DLPFC of BD, indicating the interaction among these pathological processes in mood disorders with PDCD6IP playing a key role. In addition, a number of suppressed biological processes related to neuronal projection were observed, including distal axon, dendrite and postsynaptic density. Overlapping proteins involved both in tissue remodeling and neuronal projection included DBN1, MYH9 and DBNL (Supplementary Table 7). It is of interest to note that in the DLPFC of BD, we also observed several downregulated GO terms related to energy metabolism, including the mitochondrial matrix, mitochondrial envelope and nucleotide metabolic process. Indeed, similar mitochondrial dysfunction and abnormalities of brain energy metabolism were found both in BD and MDD [47–50]. In addition, abnormalities in the mitochondrial structure and distribution were identified in the prefrontal cortex and in fibroblast cells obtained from BD patients [51]. One may presume that the effect of rTMS applied to the DLPFC of BD patients [5] is based upon upregulating metabolism.

### No significantly functional cluster changes found in the ACC of BD patients compared with their respective controls

In this study we found that, compared with the DLPFC of BD, or with the ACC of MDD, there were much less, or no significant, proteomic changes in the ACC of BD patients. A previous transcriptome sequencing and genome-wide association analysis found actin cytoskeleton remodeling is activated in the ACC of BD [52], indicating that different omics techniques may reveal different pathological mechanisms.

It is noteworthy that although our study revealed the change of thousands of proteins in the DLPFC and ACC of MDD and BD patients, certain peptides or receptor proteins that our group observed to change significantly in these two brain areas and associated with these two mood disorders have not been identified in present proteomics studies. Examples are corticotropin-releasing hormone and its receptors, oxytocin and its receptors, estrogen receptors, and androgen receptors [53–55]. This may be due to the fact that proteomics techniques detect larger, high-abundance proteins, while molecular assay techniques effectively measure small peptides and/or proteins present in low abundance. This reminds us of the necessity to use complementary techniques for future mechanism studies in order to observe the pathological changes of the full range of peptide/protein expression. It should be kept in mind that the proteomic changes we found are ‘snapshots’ while the mood disorder pathological processes in the brain are progressive and dynamic. In the future, continuous proteomic analysis of brain samples from patients at different stages of the disease may reveal the original cause of the disease. Furthermore, quantitative immunostaining and/or western blotting of the specific key proteins (see Supplementary Table 7) in the DLPFC and ACC of BD and MDD patients are of importance to further elucidate the neuropathogenesis.

### Limitations of our study

There are some limitations to our study that are inherent to the use of postmortem tissue

Medication is one of the potential confounding factors in postmortem studies that cannot possibly be matched for in an ideal way. Therefore, we rely, additionally, on animal experiments. Historically, we have *not* found that medications play a significant role affecting our results or conclusions in our postmortem studies on MDD and BD [4, 56, 57]. In addition, a previous study of animal models found that selective serotonin reuptake inhibitors (SSRIs), e.g., fluoxetine, administration in overfed rats improved mitochondrial respiratory chain activity and oxidative balance, as well as the transcription of genes employed in mitochondrial biogenesis [58]. Moreover, a recent proteomic study found that chronic fluoxetine treatment increased the energy metabolism towards the TCA cycle and oxidative phosphorylation in rat hippocampal non-synaptic mitochondria [59]. We observed significantly decreased energy metabolism in the DLPFC of MDD patients with or without antidepressant treatment. The medication influence on the key proteins analyzed by the CTD database (http://ctdbase.org/) showed that clozapine may lead to increased expression of PFKP; haloperidol may lead to increased expression of PFKP and PFN1 (Supplementary Table 7). In this study, only one MDD patient showed history of using clozapine, and only two MDD patients used haloperidol (Supplementary Table 8), while the expression of PFKP (in the DLPFC of MDD) and PFN1 (in the ACC of MDD patients) were lower than in their respective controls. Therefore, if these compounds interfered with our measurements, this would have led to an underestimation of the decreased energy metabolism we observed.

In the present study, primary neurological disorders or psychiatric disorders other than MDD or BD, and alcohol abuse, were excluded. However, our elderly brain donors had a variety of multiple physical diseases (and their treatments) that might act as confounders. The great individual variety of these disorders and treatments will, however, most probably not have confounded our data in any systematic way. In addition, it should be noted that physical illnesses can cause depression and may also cause modifications of genetic expression that are not necessarily in the direction of the study findings. In our study, depression was excluded in the controls, and the BD or MDD patients had chronic - if not lifelong- mood disorder. Therefore, a relationship between the mood disorder and the physical illness of the elderly donors is not probable. Physical illness, such as pneumonia, can also influence the way of dying of the donors. This is the reason why we have strictly matched for the CSF-pH value which is a parameter for the agonal state of dying [12, 60].

## Conclusion

Overall, our work investigated the proteomics profiling systematically in the DLPFC and ACC of MDD and BD and provides a rich resource for future study on the pathology of different mood disorders. Our results suggest the heterogeneity of different brain regions and of different mood disorders, which may relate to different pathological progression. Based on our results, further studies of specific area changes in specific mood disorders based upon detailed brain function evaluation would provide more information regarding the mechanism of symptoms of different mood disorders.

## Supplementary information

supplementary material
