# Supplementary material for: Distinct proteomic profiles in prefrontal subareas of elderly major depressive disorder and bipolar disorder patients
Source: Transl Psychiatry. 2022 Jul 11;12:275. doi: 10.1038/s41398-022-02040-7 (PMC9276790; doi:10.1038/s41398-022-02040-7)
Supplement: Supplementary file 1 — supplementary material [file 41398_2022_2040_MOESM1_ESM.docx]

**­­­Supplementary material contents：**

| **Contents** | **Pages** |
| --- | --- |
| Supplementary material and methods | P 2-6 |
| Supplementary result | P 6-7 |
| Supplementary Table 1·1 | P 8-12 |
| Supplementary Table 1·2 | P 13-17 |
| Supplementary Table 2 | P 18-19 |
| Supplementary Table 3 | P 20 |
| Supplementary Table 4 | P 21-23 |
| ­­Supplementary Table 5 | P 24-27 |
| Supplementary Table 6 | P 28 |
| Supplementary Table 7 | P 29-31 |
| Supplementary Table 8 | P 32-34 |
| Supplementary Figure 1 | P 35 |
| Supplementary Figure 2 | P 36 |
| Supplementary Figure 3 | P 37 |
| Supplementary Figure 4 | P 38 |
| Supplementary Figure 5 | P 39 |
| Supplementary Figure 6 | P 40 |
| Reference | P 41-42 |

**Supplementary Materials and Methods**

**1. Brain material**

Cryostat sections of the DLPFC (dorsolateral prefrontal cortex) and ACC (anterior cingulate cortex) were cut with 20 μm thickness, then snap-frozen at -80 ^o^C until use. For every two consecutive sections, one was stored at -20 ^0^C for immunohistochemical staining (see below); and the other, which contained some 1 mg brain tissue and 100 μg protein, was kept in a 1·5 ml Eppendorf tube at -80 ^o^C for the proteomics study. Gray matter contained all 6 layers of the neocortex, and white matter was also present.

**2. Reagents for experiments**

Sodium Deoxycholate (SDC, Cat no: 30970, Stock solution: 5% in Milli-Q®), Dithiothreitol (DTT, Cat no: D8255, Stock solution: 100 mM in Milli-Q), Iodoacetamide (IAA, Cat no: I6125, Stock solution: 200 mM in Milli-Q), 250 mM 4-(2-Hydroxyethyl)-1-piperazinepropanesulfonic acid buffer (EPPS, Cat no: E9502, pH 8·2 and pH 7·8) and MS-SAFE protease and phosphatase inhibitor (Cat no: MSSAFE) were purchased from Sigma-Aldrich. IAA was always prepared fresh because of its light sensitivity. Pierce bicinchoninic acid (BCA) protein assay kit (BCA Assay, Cat.no: 23225), TMT10plex Isobaric Label Reagent Set plus TMT11-131C Label Reagent (Cat. No A37725) and trypsin (Cat no: 20233) were purchased from Thermo Fisher Scientific. Lysyl endopeptidase (LysC, Cat no: 125-02541) was purchased from Wako.

**3.** **Quantification of the percentages of grey matter and white matter**

Thionine staining and a computerized image system (Image-Pro Plus version 6·3) were used to determine whether or not the percentages of grey matter and white matter were different among the groups. Cryostat sections of ACC or DLPFC were taken out from the -80 ^o^C freezer and fixed for 10 min in 10% formalin/phosphate buffered saline (PBS) solution at room temperature. After three rinses in PBS, sections were incubated in 0·5% thionine solution for 5-10 min. Differentiation was performed by dipping in distilled water, and an ascending ethanol series. Sections were finally cleared in xylene and coverslipped using Entellan. The percentages of grey matter and white matter were calculated using a computerized image system Image-Pro Plus version 6·3. The system was connected to a JVC 3CCD camera equipped with a Zeiss Axioskop microscope (Plan-Neofluar). Area determination was performed as Supplementary Figure1 shows: in each section to be analyzed, the whole area was loaded into the system and displayed on the image analysis monitor. Grey matter and white matter were outlined manually. Subsequently, the percentages of grey matter and white matter were determined automatically by the image analysis system. The percentage of grey matter was significantly higher (73%) than that of white matter (27%) in the DLPFC and ACC samples of MDD, BD and their respective controls (Supplementary figure 1A-B). The ratios of grey matter/white matter were around 2·7 both in the DLPFC and the ACC of MDD, BD and their respective controls without significant differences (Kruskal-Wallis test: DLPFC, p = 0·927; ACC, p = 0·869).

**4. Protein concentration determination**

Protein concentrations were determined using the BCA assay. Briefly, working reagent (WR) was prepared firstly by combination of 50 ml reagent A and 1 ml reagent B in the kit. Next, 0·1 ml sample and 2 ml WR were added into a test tube and well mixed. The tube was incubated for 30 min at 37 ^0^C and then cooled. Finally, with the spectrophotometer set to 562 nm, absorbance of all samples was measured and the concentration was then determined.

**5.** **Chloroform/Methanol protein precipitation**

As previously described (1), 4 volumes of methanol followed by 1 volume of chloroform were added to the reduced and alkylated sample and mixed by vortexing. Afterwards, 3 volumes Milli-Q water were added and mixed by vortexing. The samples were centrifuged for 5 min at 14000 g. The upper phase was removed and discarded. 3 volumes of methanol were added to the lower phase and the sample was centrifuged for 5 min at 14000 g. The supernatant was removed and the protein pellet was air-dried for 10 min.

**6. TMT labeling**

Peptides were labeled with TMT-11plex reagents (Thermo Fisher Scientific) according to the manufacturer’s protocol. In brief, 0·2 mg TMT label reagents were dissolved in 20 μL of anhydrous acetonitrile and added to 25 μl peptide sample (10-25 μg protein digest) this was incubated for 1 h at RT. 1 μl of a 5% hydroxylamine solution was added to the sample and incubated for 15 min to terminate the reaction.

**7. Nanoflow LC-MS/MS**

Nanoflow LC−MS/MS was performed on an Easy nLC 1200 system (Thermo Fisher Scientific) coupled to an Orbitrap Fusion Lumos Tribrid mass spectrometer (Thermo Fisher Scientific), operating in positive mode and equipped with a nanospray source. Peptide mixtures were trapped on a ReproSil C18 reversed-phase column (Dr Maisch; column dimensions 1·5 cm × 100 μm, packed in-house) at a flow rate of 2 μl/min. Peptide separation was performed on ReproSil C18 reversed-phase column (Dr Maisch; column dimensions 50 cm × 75 μm, packed in-house) using a linear gradient from 0 to 80% B (A = 0·1% FA; B = 80% (v/v) AcN, 0·1% FA) in 120 min and at a constant flow rate of 250 nl/min. The column eluent was directly sprayed into the ESI source of the mass spectrometer. All mass spectra were acquired in profile mode. The resolution in MS1 mode was set to 70000 (AGC: 3E6), the m/z range 350−1700. Fragmentation of precursors was performed in data-dependent mode using the multinotch SPS MS3 reporter ion-based quantification method.

**8. Detailed settings of the Mascot search algorithm**

The peptide tolerance was set to 20 ppm and the fragment ion tolerance was set to 0·5 Da. The reporter ion tolerance was set to 0·003 Da. A maximum number of 2 missed cleavages by trypsin were allowed and carbamidomethylated cysteine and oxidized methionine were set as fixed and variable modifications, respectively. Typical contaminants were omitted from the output tables. Protein ratios were calculated from the abundances of the reporter ions over the 11 quantitation channels. For correct matching between different runs, an internal reference standard (IRS) channel was used. The IRS sample was composed of a fraction of all samples mixed in a 1:1 ratio to obtain a sample of ‘average composition’. Normalization of reporter ion signals from all runs was performed using in-house built software tools.

Researcher was blinded to the group allocation during the experiment until the start of statistical analysis.

**9. Statistical and Bioinformatic analysis**

SPSS was used for analysis of grey matter/white matter ratio, and for comparing the expression levels of specific cell type markers. The Kolmogorov-Smirnov test was used to test the normality distribution of the ratios of grey matter/white matter among the MDD, BD and their respective controls. Kruskal-Wallis test was used to detect the differences among multiple groups, and one-way ANOVA was used to compare the expression levels of specific cell type markers. The Bonferroni test was used as a post-hoc test. Student’s t-test was used on differential expression analysis of protein data to determine the proteomic changes between the MDD or BD patients and their respective controls. Functional implications of the differentially expressed proteins were further explored by gene ontology (GO) enrichment analysis. The identified proteins were classified in Perseus and by GO terms, based on the molecular functions of given protein, cellular component, and/or biological process. The enrichment analysis was performed using the online UniProt database (https://www.uniprot.org/). The enriched GO terms were sorted according to their enrichment fold. Rstudio (v 3.3.0) was used to analyze the relationship of specific proteins and GO terms, and to calculate the z-score of GO terms. An inhibited or activated status was considered when z-score < -2 or z-score > 2, respectively. The specific functions of given proteins were identified by the UniProt database.

**Supplementary Results**

When MDD DLPFC (n=16) was compared with BD DLPFC (n=5), there were 105 significantly differentially expressed proteins (DEPs), and when MDD ACC (n=12) was compared with BD ACC (n=7), there were 177 significantly DEPs (Supplementary Figure 6A and 6E).

A total of 11 GO terms were significantly activated in the DLPFC of MDD compared with the DLPFC of BD by the DEPs (Supplementary Table 8, Supplementary Figure 6C) via GO analysis, among which 6 GO terms were associated with synaptic function, including neuron to neuron synapse, regulation of trans-synaptic signaling and modulation of chemical synaptic transmission. We further subjected the protein expression changes to Goplot with the purpose of elucidating the relationship between specific proteins and changed GO terms. Multiple upregulated key proteins related to synaptic function were found in the DLPFC of MDD compared with the DLPFC of BD, which included SYN3, PDLIM5 and PRNP. These molecules have been found to be involved in neurotransmitter release and synaptic plasticity (details see Supplementary Table 7). We observed that neuronal projection, which includes GO terms such as neuron to neuron synapse, dendritic tree and postsynaptic density, was suppressed in the DLPFC of BD patients (See page 11 line 13-14), but not in the DLPFC of MDD patients (See page 9 line 15-16), when compared with their respective controls. This observation coupled with the relatively activated synaptic function in the DLPFC of MDD compared with the DLPFC of BD patients further supports our conclusions.

A total of 89 GO terms were significantly suppressed in the ACC of MDD compared with the ACC of BD as determined by the DEPs (Supplementary Table 8) by GO analysis, among which the most significantly suppressed functional cluster was vesicle function, including Golgi vesicle transport, regulation of vesicle-mediated transport, regulation of neurotransmitter transport and vesicle localization (Supplementary Figure 6G). Multiple downregulated key proteins related to vesicle function were found in the ACC of MDD compared with the ACC of BD, which included STXBP1, GOLGA2 and VPS4A (Supplementary Figure 6H). These molecules have been found to be involved in vesicle functions such as neurotransmitter secretion and transport (Supplementary Table 7). Vesicle formation is an important part of neurotransmitter transport, which is a key process of synaptic function. Presynaptic components are predominantly synthesized in the neuronal soma, packaged into vesicles and actively transported along the axon to sites of presynaptic biogenesis (2, 3). As we observed that 3 GO terms related to synaptic function, i.e. axon, axon part and postsynapse, were suppressed in the ACC of MDD patients (See page 10 line 2-3), but not in the ACC of BD patients (See page 12 line 19-21 and page 13 line 1-2), when compared with their respective controls, the relatively lower vesicle function in the ACC of MDD compared with the ACC of BD patients further supports our conclusions.

**Table S1.1 Clinical information of major depressive disorder (MDD) patients, bipolar disorder (BD) patients and relative control group: dorsolateral prefrontal cortex (DLPFC)**

| **NBB** | **Group** | **Sex** | **Age (y)** | **PMD (hr:min)** | **COD** | **MOD** | **CSF**  **pH** | **BW (g)** | **Medication** | **Suicide attempt** | **Cause of death** |
| --- | --- | --- | --- | --- | --- | --- | --- | --- | --- | --- | --- |
| **MDD collection** | | | | | | | | | | | |
| 2006-011 | MDD | F | 60 | 04:20 | 16:10 | 1 |  | 1074 | Haloperidol | no | Legal euthanasia |
| 2014-060 | MDD | F | 62 | 11:40 | 16:15 | 10 | 6.18 | 1155 | no | yes | Escitalopram |
| 2015-028 | MDD | F | 66 | 07:55 | 14:35 | 3 |  | 1165 | Quetiapine; Mirtazapine | yes | Legal euthanasia |
| 2012-097 | MDD | F | 73 | 05:45 | 15:30 | 9 | 6.7 | 1205 | no | no | Mirtazapine |
| 2012-081 | MDD | F | 80 | 06:40 | 16:30 | 8 | 6.62 | 1237 | Amitriptyline | no | Sudden death |
| 2016-112 | MDD | F | 77 | 8:40 | 11:20 | 11 | 6.77 | 1120 | SSRI | yes | Legal euthanasia |
| 2009-106 | MDD | F | 84 | 08:45 | 6:30 | 12 | 6 | 1140 | Lorazepam | no | Cachexia and dehydration from bladder carcinoma |
| 2016-024 | MDD | F | 88 | 10:00 | 9:00 | 2 | 6.26 | 1160 | Oxazepam; Clozapine | no | Burns, dehydration |
| 2008-076 | MDD | F | 91 | 05:20 | 9:05 | 8 | 6.53 | 1163 | SSRI | no | Cachexia and dehydration by pneumonia and renal insufficiency |
| 2008-031 | MDD | F | 93 | 04:20 | 4:55 | 3 | 6.8 | 1023 | SSRI | yes | Pneumonia |
| 2011-051 | MDD | F | 100 | 05:50 | 10:50 | 6 | 6.4 | 990 | Haloperidol | no | Natural death |
| 2017-032 | MDD | M | 48 | 12:45 | 10:15 | 2 | 6.76 | 1350 | Tranylcypromine | yes | Legal euthanasia |
| 2011-058 | MDD | M | 83 | 10:40 | 5:00 | 7 | 6.5 | 1200 | Diazepam | no | Acute heart failure |
| 2007-033 | MDD | M | 88 | 06:37 | 21:15 | 5 | 6.26 | 1175 | Oxazepam | no | Multiple epileptic seizures |
| 2013-085 | MDD | M | 89 | 04:35 | 3:15 | 11 | 6.67 | 1225 | Paroxetine; Oxazepam | yes | Metastasized prostate carcinoma |
| 2007-060 | MDD | M | 93 | 06:00 | 21:10 | 9 | 6.37 | 1369 | Dexamphetamine | no | Cachexia in combination with CVA or failure of renal functions |
| Median |  |  | 83.5 | 6:39 |  |  | 6.52 | 1164 |  |  |  |
| Mean±SD |  |  | 79.7±14.4 | 7:30±  2:40 |  |  | 6.49±0.25 | 1172±  99.5 |  |  |  |
| 2015-055 | CTR | F | 72 | 06:50 | 16:00 | 6 | 7.22 | 1165 | no | no | Euthanasia, metastatic ovarian cancer and ileus |
| 2015-087 | CTR | F | 75 | 09:10 | 11:10 | 10 | 6.57 | 1305 | no | no | Legal euthanasia |
| 2015-027 | CTR | F | 76 | 04:45 | 5:50 | 3 | 6.4 | 1140 | Haloperidol | no | Adenocarcinoma |
| 2012-059 | CTR | F | 78 | 04:35 | 22:40 | 6 | 6.41 | 1176 | Temazepam | no | Bronchopneumonia |
| 2015-034 | CTR | F | 82 | 07:45 | 2:00 | 4 | 5.97 | 1318 | no | no | Lung cancer, pneumonia |
| 2011-021 | CTR | F | 85 | 07:05 | 13:40 | 3 | ? | 1007 | no | no | Terminal renal insufficiency |
| 2011-090 | CTR | F | 85 | 08:25 | 13:05 | 10 | 6.51 | 1240 | Temazepam | no | Dehydration and cachexia |
| 2012-086 | CTR | F | 88 | 05:25 | 3:35 | 8 | 6.33 | 1010 | no | no | Abdominal haematoma |
| 2013-010 | CTR | F | 89 | 06:35 | 2:40 | 2 | 6.73 | 1139 | Haloperidol | no | Heart failure and dehydration |
| 2015-016 | CTR | F | 95 | 07:05 | 4:15 | 2 | 5.89 | 1010 | no | no | Liver abscess with sepsis |
| 2014-063 | CTR | F | 93 | 07:35 | 15:15 | 10 | 6.27 | 1025 | no | no | Heart failure |
| 2014-069 | CTR | M | 73 | 04:25 | 5:05 | 11 | 7 | 1285 | Haloperidol | no | Pneumonia |
| 2012-101 | CTR | M | 80 | 04:25 | 17:40 | 9 | 6.59 | 1429 | no | no | Legal euthanasia |
| 2012-092 | CTR | M | 90 | 05:45 | 10:50 | 8 | 6.35 | 1215 | no | no | Unknown |
| 2009-096 | CTR | M | 92 | 08:25 | 1:45 | 12 | 6.14 | 1117 | no | no | Heart failure |
| 2015-033 | CTR | M | 93 | 07:40 | 9:15 | 4 | 6.2 | 1155 | no | no | Heart failure |
| Median |  |  | 85.0 | 6:58 |  |  | 6.40 | 1160 |  |  |  |
| Mean±SD |  |  | 84.1±7.7 | 6:37±  1:33 |  |  | 6.44±0.36 | 1171±  124.1 |  |  |  |
| P value |  |  | 0.52 | 0.45 | 0.89 | 0.94 | 0.48 | 0.90 |  |  |  |
| **BD collection** | | | | | | | | | | | |
| 2015-107 | BD | F | 77 | 06:30 | 2:50 | 12 | 6.5 | 1115 | no | no | Renal insufficiency |
| 2014-041 | BD | F | 79 | 08:00 | 16:45 | 7 | 6.31 | 990 | Lithium; Quetiapine | no | Renal insufficiency |
| 2006-075 | BD | F | 80 | 09:30 | 9:30 | 10 | 6.33 | 1140 | SSRI | no | Relapsing bronchitis |
| 2015-101 | BD | F | 92 | 07:25 | 9:30 | 11 | 6.7 | 1025 | Quetiapine; Nortriptyline | no | Atrial fibrillations, infection |
| 2012-110 | BD | M | 87 | 03:15 | 23:00 | 10 | 6.39 | 1285 | no | no | Pneumonia |
| Median |  |  | 80.0 | 7:25 |  |  | 6.39 | 1115 |  |  |  |
| Mean±SD |  |  | 83.0±6.3 | 6:56±  1:03 |  |  | 6.45±0.16 | 1111±  115.3 |  |  |  |
| 2014-029 | CTR | F | 78 | 07:10 | 15:05 | 5 | 6.32 | 1120 | Oxazepam | no | Legal euthanasia |
| 2014-053 | CTR | F | 80 | 07:04 | 20:30 | 9 | 6.2 | 1450 | Haloperidol | no | Legal euthanasia |
| 2011-114 | CTR | F | 81 | 05:30 | 8:20 | 11 | 6.77 | 1192 | no | no | Respiratory insufficiency by advanced COPD |
| 2014-020 | CTR | F | 92 | 06:35 | 12:45 | 4 | 6.12 | 1305 | no | no | Heart failure |
| 2013-013 | CTR | M | 89 | 06:50 | 23:25 | 2 | 6.23 | 1185 | no | no | Urosepsis |
| Median |  |  | 81 | 6:50 |  |  | 6.23 | 1192 |  |  |  |
| Mean±SD |  |  | 84.0±6.1 | 6:38±  0:40 |  |  | 6.33±0.26 | 1250±  129.9 |  |  |  |
| P value |  |  | 0.68 | 0.55 | 0.87 | 0.27 | 0.22 | 0.10 |  |  |  |

Abbreviations: BW, Brain weight; CSF, cerebrospinal fluid; COD, clock time of death; CTR, control; F, female; M, male; MDD, major depressive disorder; BD, bipolar disorder; MOD, month of death; NBB, Netherlands Brain Bank; PMD, postmortem delay; SSRI, selective serotonin reuptake inhibitor.

**Table S1.2 clinical information of major depressive disorder (MDD) patients, bipolar disorder (BD) patients and relative control group: anterior cingulate cortex (ACC)**

| **NBB** | **Group** | **Sex** | **Age (y)** | **PMD (hr:min)** | | **COD** | **MOD** | **CSF**  **pH** | **BW (g)** | **Medication** | **Suicide attempt** | **Cause of death** |
| --- | --- | --- | --- | --- | --- | --- | --- | --- | --- | --- | --- | --- |
| **MDD collection** | | | | | | | | | | | | |
| 2014-060 | MDD | F | 62 | 11:40 | | 16:15 | 10 | 6.18 | 1155 | Escitalopram | yes | Suicide |
| 2015-028 | MDD | F | 66 | 07:55 | | 14:35 | 3 |  | 1165 | Quetiapine; Mirtazapine | yes | Legal euthanasia |
| 2012-097 | MDD | F | 73 | 05:45 | | 15:30 | 9 | 6.7 | 1205 | Mirtazapine | no | Legal euthanasia |
| 2012-081 | MDD | F | 80 | 06:40 | | 16:30 | 8 | 6.62 | 1237 | Amitriptyline | no | Sudden death |
| 2010-072 | MDD | F | 83 | 04:05 | | 14:25 | 7 | 6.75 | 1066 | no | yes | Legal euthanasia |
| 2016-024 | MDD | F | 88 | 10:00 | | 9:00 | 2 | 6.26 | 1160 | Oxazepam; Clozapine | no | Burns, dehydration |
| 2016-112 | MDD | F | 77 | 8:40 | | 11:20 | 11 | 6.77 | 1120 | SSRI | yes | Legal euthanasia |
| 2011-051 | MDD | F | 100 | 05:50 | | 10:50 | 6 | 6.4 | 990 | Haloperidol | no | Natural death |
| 2014-012 | MDD | M | 68 | 08:55 | | 5:45 | 3 | 6.82 | 1510 | Paroxetine; Oxazepam | yes | Sudden death possibly related to panlobular emphysema, pneumonia or cardiac |
| 2015-096 | MDD | M | 69 | 04:40 | | 20:10 | 11 | 6.63 | 1145 | SSRI | yes | Ascites, cirrhosis of the liver, multiple organ dysfunction syndrome (MODS) |
| 2011-058 | MDD | M | 83 | 10:40 | | 5:00 | 7 | 6.5 | 1200 | Diazepam | no | Acute heart failure |
| 2013-085 | MDD | M | 89 | 04:35 | | 3:15 | 11 | 6.67 | 1225 | no | no | unknown |
| Median |  |  | 78.5 | | 7:18 |  |  | 6.63 | 1163 |  |  |  |
| Mean±SD |  |  | 78.2±11.1 | | 7:27±  2:33 |  |  | 6.57±0.21 | 1182±  124.4 |  |  |  |
| 2014-043 | CTR | F | 60 | | 08:10 | 17:15 | 7 | 6.58 | 1310 | no | no | Metastasized mamma carcinoma |
| 2012-049 | CTR | F | 70 | | 07:35 | 4:45 | 5 | 6.03 | 1188 | no | no | Cachexia by endstage pancreas carcinoma |
| 2004-057 | CTR | F | 81 | | 06:40 | 13:10 | 8 | 7.16 | 1161 | no | no | Legal euthanasia |
| 2011-049 | CTR | F | 83 | | 04:40 | 22:20 | 6 | 6.04 | 1130 | no | no | Ileus with pancreatic cancer |
| 2011-090 | CTR | F | 85 | | 08:25 | 13:05 | 10 | 6.51 | 1240 | Temazepam | no | Dehydration and cachexia |
| 1993-035 | CTR | F | 89 | | 04:20 | 6:15 | 3 | 6.68 | 1068 | no | no | Heart failure |
| 2012-001 | CTR | F | 89 | | 05:40 | 2:10 | 1 | 6.75 | 943 | unknown | no | Pneumonia and cardiac decompensation |
| 2015-035 | CTR | M | 73 | | 08:00 | 16:15 | 4 | 5.37 | 1553 | no | no | Invasive fungal infection and bacterial pneumonia |
| 2002-035 | CTR | M | 79 | | 06:15 | 18:55 | 4 | 6.53 | 1322 | Temazepam | no | Acute dyspnea and dehydration. Perhaps as a result of colon carcinoma |
| 2011-017 | CTR | M | 83 | | 05:45 | 3:00 | 2 | 6.35 | 1590 | SSRI | no | Pneumonia |
| 2012-117 | CTR | F | 85 | | 06:40 | 17:50 | 11 | 6.82 | 1085 | no | no | Pneumonia |
| 2005-060 | CTR | M | 91 | | 08:00 | 7:00 | 9 | 6.26 | 1188 | no | no | Cardiac decompensation |
| Median |  |  | 83 | | 6:40 |  |  | 6.52 | 1188 |  |  |  |
| Mean±SD |  |  | 80.7±9.0 | | 6:41±  1:23 |  |  | 6.42±0.46 | 1232±  189.9 |  |  |  |
| P value |  |  | 0.38 | | 0.50 | 0.32 | 0.50 | 0.42 | 0.50 |  |  |  |
| **BD collection** | | | | | | | | | | | | |
| 2015-107 | BD | F | 77 | | 06:30 | 2:50 | 12 | 6.5 | 1115 | no | no | Renal insufficiency |
| 2014-041 | BD | F | 79 | | 08:00 | 16:45 | 7 | 6.31 | 990 | Lithium; Quetiapine | no | Renal insufficiency |
| 2015-101 | BD | F | 92 | | 07:25 | 9:30 | 11 | 6.7 | 1025 | no | no | Quetiapine; Nortriptyline |
| 2013-038 | BD | M | 72 | | 04:35 | 8:45 | 5 | 6.4 | 1294 | Lithium; SSRI | no | Dehydration and cachexia. Lithium intoxication |
| 2015-077 | BD | M | 72 | | 09:55 | 8:25 | 9 | 6.47 | 1385 | SSRI | no | Cardiac insufficiency and pulmonary infection |
| 2012-048 | BD | M | 81 | | 06:40 | 20:00 | 5 | 6.7 | 1283 | Lithium | no | Legal euthanasia |
| 2012-110 | BD | M | 87 | | 03:15 | 23:00 | 10 | 6.39 | 1285 | no | no | Pneumonia |
| Median |  |  | 79 | | 6:40 |  |  | 6.47 | 1283 |  |  |  |
| Mean±SD |  |  | 80.0±7.4 | | 6:37±  2:12 |  |  | 6.50±0.15 | 1197±  152.2 |  |  |  |
| 2012-117 | CTR | F | 85 | | 06:40 | 17:50 | 11 | 6.82 | 1085 | no | no | unknown |
| 2010-038 | CTR | F | 79 | | 10:30 | 8:00 | 4 | 6.3 | 1148 | no | no | Cardiac insufficiency |
| 2014-063 | CTR | F | 93 | | 07:35 | 15:15 | 10 | 6.27 | 1025 | no | no | Heart failure |
| 2014-069 | CTR | M | 73 | | 04:25 | 5:05 | 11 | 7 | 1285 | Haloperidol | no | Pneumonia |
| 2010-013 | CTR | M | 70 | | 06:15 | 17:15 | 2 | 6.45 | 1502 | Paroxetine | no | Unknown. Acute myocardial infarction or pulmonary embolism were mentioned as possibilities |
| 2001-033 | CTR | M | 75 | | 06:20 | 6:10 | 3 | 6.18 | 1145 | no | no | unknown |
| 2009-001 | CTR | M | 88 | | 04:43 | 19:47 | 1 | 6.17 | 1418 | no | no | Gastro-intestinal bleeding |
| Median |  |  | 79 | | 6:20 |  |  | 6.30 | 1148 |  |  |  |
| Mean±SD |  |  | 80.4±8.5 | | 6:38±  2:02 |  |  | 6.46±0.33 | 1230±  177.6 |  |  |  |
| P value |  |  | 0.92 | | 0.83 | 0.92 | 0.16 | 0.36 | 0.69 |  |  |  |

Abbreviations: BW, Brain weight; CSF, cerebrospinal fluid; COD, clock time of death; CTR, control; F, female; M, male; MDD, major depressive disorder; BD, bipolar disorder; MOD, month of death; NBB, Netherlands Brain Bank; PMD, postmortem delay; SSRI, selective serotonin reuptake inhibitor.

**Table S2: Significantly overrepresented GO (Gene Ontology) terms in the dorsolateral prefrontal cortex (DLPFC) of major depressive disorder (MDD) patients**

| **GO term** | **adj_pval** | **z-score** |
| --- | --- | --- |
| nucleoside triphosphate metabolic process | 7.85E-08 | -3.0532901 |
| mitochondrial matrix | 1.92E-09 | -2.9824045 |
| nucleoside triphosphate biosynthetic process | 0.00048741 | -2.6457513 |
| mitochondrial membrane | 9.67E-09 | -2.4748737 |
| ATP biosynthetic process | 0.00118856 | -2.4494897 |
| mitochondrial nucleoid | 6.92E-07 | -2.4494897 |
| nucleoid | 6.92E-07 | -2.4494897 |
| purine nucleoside triphosphate biosynthetic process | 0.00171863 | -2.4494897 |
| purine ribonucleoside triphosphate biosynthetic process | 0.00166863 | -2.4494897 |
| ribonucleoside triphosphate biosynthetic process | 0.00198591 | -2.4494897 |
| nucleoside phosphate metabolic process | 5.89E-06 | -2.3570226 |
| nucleotide metabolic process | 5.15E-06 | -2.3570226 |
| purine-containing compound metabolic process | 6.53E-07 | -2.3570226 |
| nucleoside phosphate biosynthetic process | 0.00241402 | -2.3333333 |
| ATP metabolic process | 2.79E-06 | -2.3094011 |
| purine nucleotide metabolic process | 1.27E-06 | -2.1828206 |
| purine ribonucleotide metabolic process | 5.65E-07 | -2.1828206 |
| ribonucleotide metabolic process | 8.22E-07 | -2.1828206 |
| ribose phosphate metabolic process | 1.33E-06 | -2.1828206 |
| purine nucleoside triphosphate metabolic process | 2.51E-07 | -2.1380899 |
| purine ribonucleoside triphosphate metabolic process | 1.95E-07 | -2.1380899 |
| ribonucleoside triphosphate metabolic process | 2.42E-07 | -2.1380899 |
| succinyl-CoA metabolic process | 7.61E-06 | -2.1380899 |
| mitochondrial envelope | 2.68E-08 | -2.1320072 |
| nucleotide biosynthetic process | 0.00779793 | -2.1213203 |
| purine nucleotide biosynthetic process | 0.00172018 | -2.1213203 |
| purine ribonucleotide biosynthetic process | 0.00111245 | -2.1213203 |
| purine-containing compound biosynthetic process | 0.00223888 | -2.1213203 |
| ribonucleotide biosynthetic process | 0.00148329 | -2.1213203 |
| ribose phosphate biosynthetic process | 0.00172018 | -2.1213203 |
| organophosphate biosynthetic process | 0.00066684 | -3.2071349 |
| carbohydrate derivative biosynthetic process | 0.00484662 | -3.0508511 |
| response to metal ion | 1.67E-05 | -2.8867513 |
| response to calcium ion | 1.47E-05 | -2.8401878 |
| response to inorganic substance | 1.80E-05 | -2.8401878 |
| small molecule catabolic process | 2.82E-05 | -2.4961509 |
| ERK1 and ERK2 cascade | 0.00064295 | -2.3333333 |
| regulation of ERK1 and ERK2 cascade | 0.00042555 | -2.3333333 |
| antibiotic metabolic process | 0.00517005 | -2.236068 |
| dicarboxylic acid metabolic process | 0.00085711 | -2.236068 |
| positive regulation of ERK1 and ERK2 cascade | 0.0002072 | -2.1213203 |

**Table S3: Significantly overrepresented GO (Gene Ontology) terms related to synaptic function in the dorsolateral prefrontal cortex (DLPFC) of major depressive disorder (MDD) patients**

| **GO term** | **adj_pval** | **z-score** |
| --- | --- | --- |
| dendritic spine | 0.0002563 | -1.8898224 |
| neuron spine | 0.00027534 | -1.8898224 |
| postsynapse | 6.53E-07 | -1.7320508 |
| dendrite | 0.00020163 | -1.6035675 |
| dendritic tree | 0.00020837 | -1.6035675 |
| asymmetric synapse | 0.00016168 | -1.2649111 |
| neuron to neuron synapse | 0.00027204 | -1.2649111 |
| postsynaptic density | 0.00014638 | -1.2649111 |
| postsynaptic specialization | 0.00025993 | -1.2649111 |
| regulation of trans-synaptic signaling | 0.00149358 | -1.2649111 |
| anterograde trans-synaptic signaling | 0.00600403 | -1.1547005 |
| chemical synaptic transmission | 0.00600403 | -1.1547005 |
| synaptic signaling | 0.0069746 | -1.1547005 |
| trans-synaptic signaling | 0.0065445 | -1.1547005 |

**Table S4: Significantly overrepresented GO (Gene Ontology) terms in the anterior cingulate cortex (ACC) of major depressive disorder (MDD) patients**

| **GO term** | **adj_pval** | **z-score** |
| --- | --- | --- |
| cadherin binding | 5.53E-07 | -4.1576092 |
| cytokinesis | 2.18E-06 | -3.904344 |
| adherens junction | 6.88E-12 | -3.7796447 |
| anchoring junction | 1.35E-11 | -3.7796447 |
| focal adhesion | 1.89E-12 | -3.7796447 |
| oxidoreductase activity | 3.37E-08 | -3.6181361 |
| organic acid catabolic process | 1.57E-06 | -3.5282114 |
| small molecule catabolic process | 2.49E-05 | -3.5 |
| cell-substrate adherens junction | 2.22E-12 | -3.4 |
| cell-substrate junction | 2.75E-12 | -3.4 |
| regulation of cellular component size | 1.03E-05 | -3.3565856 |
| midbody | 2.44E-06 | -3.3166248 |
| cell adhesion molecule binding | 6.92E-06 | -3.2998316 |
| actin cytoskeleton organization | 1.96E-07 | -3.2659863 |
| carboxylic acid catabolic process | 1.57E-06 | -3.2071349 |
| GTP binding | 4.99E-05 | -3.2071349 |
| guanyl nucleotide binding | 8.67E-05 | -3.2071349 |
| guanyl ribonucleotide binding | 8.67E-05 | -3.2071349 |
| nucleoside binding | 7.38E-05 | -3.2071349 |
| purine nucleoside binding | 5.91E-05 | -3.2071349 |
| purine ribonucleoside binding | 5.43E-05 | -3.2071349 |
| ribonucleoside binding | 6.08E-05 | -3.2071349 |
| protein localization to cell periphery | 4.39E-08 | -3.1529631 |
| actin filament-based process | 1.58E-07 | -3.1378582 |
| microtubule-based process | 0.00019256 | -3.1304952 |
| monocarboxylic acid metabolic process | 2.26E-05 | -3.1304952 |
| granulocyte activation | 0.00287244 | -3.0508511 |
| neutrophil activation | 0.00263813 | -3.0508511 |
| neutrophil activation involved in immune response | 0.00221641 | -3.0508511 |
| neutrophil degranulation | 0.00210132 | -3.0508511 |
| neutrophil mediated immunity | 0.00268369 | -3.0508511 |
| cellular amino acid metabolic process | 0.00015562 | -3.0508511 |
| cell division | 1.10E-07 | -3 |
| regulation of anatomical structure size | 0.00010703 | -3 |
| axon | 1.44E-07 | -2.9673015 |
| cell-cell junction | 0.00370027 | -2.8867513 |
| GTPase activity | 0.0001847 | -2.8867513 |
| negative regulation of protein complex assembly | 3.54E-08 | -2.8867513 |
| positive regulation of cellular protein localization | 0.0001847 | -2.8867513 |
| protein localization to plasma membrane | 2.44E-05 | -2.8867513 |
| negative regulation of supramolecular fiber organization | 3.88E-09 | -2.8527989 |
| cell activation involved in immune response | 0.0091187 | -2.8401878 |
| leukocyte activation involved in immune response | 0.008689 | -2.8401878 |
| leukocyte degranulation | 0.00062024 | -2.8401878 |
| myeloid cell activation involved in immune response | 0.00074844 | -2.8401878 |
| cytoplasmic vesicle lumen | 3.47E-06 | -2.8401878 |
| vesicle lumen | 3.59E-06 | -2.8401878 |
| alpha-amino acid metabolic process | 2.71E-05 | -2.7136021 |
| regulated exocytosis | 2.77E-08 | -2.6940795 |
| cellular component disassembly | 0.0023007 | -2.6726124 |
| secretory granule lumen | 8.97E-06 | -2.6726124 |
| myeloid leukocyte activation | 0.00061794 | -2.6678919 |
| protein folding | 4.03E-10 | -2.6678919 |
| negative regulation of actin filament polymerization | 2.56E-06 | -2.6457513 |
| postsynapse | 1.71E-07 | -2.5354628 |
| negative regulation of protein polymerization | 4.35E-09 | -2.5298221 |
| actin filament organization | 6.35E-08 | -2.5235731 |
| myeloid leukocyte mediated immunity | 0.00029095 | -2.5 |
| regulation of actin filament-based process | 4.32E-06 | -2.5 |
| small GTPase mediated signal transduction | 0.00811357 | -2.4961509 |
| supramolecular fiber organization | 2.37E-07 | -2.4494897 |
| positive regulation of protein localization to cell periphery | 5.36E-05 | -2.4494897 |
| unfolded protein binding | 0.00272022 | -2.4494897 |
| actin filament binding | 0.00030012 | -2.3333333 |
| actin polymerization or depolymerization | 0.00039935 | -2.3333333 |
| regulation of actin filament length | 0.00013169 | -2.3333333 |
| regulation of actin polymerization or depolymerization | 0.00012625 | -2.3333333 |
| regulation of actin cytoskeleton organization | 4.29E-06 | -2.32379 |
| actomyosin structure organization | 1.57E-06 | -2.3094011 |
| protein polymerization | 5.34E-05 | -2.3094011 |
| regulation of protein polymerization | 3.83E-06 | -2.3094011 |
| regulation of cytoskeleton organization | 1.41E-08 | -2.2936586 |
| actin filament depolymerization | 0.00026143 | -2.236068 |
| regulation of actin filament depolymerization | 0.00019924 | -2.236068 |
| cell-cell adherens junction | 0.00810707 | -2.236068 |
| homotypic cell-cell adhesion | 0.00177584 | -2.236068 |
| GDP binding | 0.00112236 | -2.236068 |
| protein depolymerization | 0.00628239 | -2.236068 |
| regulation of protein complex disassembly | 0.00702474 | -2.236068 |
| regulation of protein depolymerization | 0.00187366 | -2.236068 |
| actin binding | 4.02E-06 | -2.1828206 |
| regulation of supramolecular fiber organization | 2.36E-07 | -2.1828206 |
| regulation of cellular protein localization | 4.65E-05 | -2.1828206 |
| regulation of actin filament organization | 8.10E-07 | -2.1380899 |
| axon part | 6.08E-05 | -2.1380899 |
| protein kinase binding | 0.00980682 | -2.1380899 |
| actin filament polymerization | 0.00072948 | -2.1213203 |
| regulation of actin filament polymerization | 0.00033742 | -2.1213203 |
| cellular amino acid catabolic process | 5.61E-05 | -2.1213203 |
| regulation of protein localization to cell periphery | 3.09E-05 | -2.1213203 |
| protein localization to membrane | 6.31E-10 | -2.116951 |
| viral life cycle | 0.00079786 | -2.1105794 |

**Table S5: Significantly overrepresented GO (Gene Ontology) terms in the dorsolateral prefrontal cortex (DLPFC) of bipolar disorder (BD) patients**

| **GO term** | **adj_pval** | **z-score** |
| --- | --- | --- |
| cadherin binding | 6.81E-08 | -4.6 |
| focal adhesion | 7.17E-07 | -4 |
| microtubule-based process | 3.56E-05 | -3.8729833 |
| anchoring junction | 5.03E-06 | -3.7416574 |
| adherens junction | 1.80E-05 | -3.6055513 |
| cell adhesion molecule binding | 6.67E-06 | -3.6055513 |
| cell-substrate junction | 8.67E-07 | -3.6055513 |
| actin filament-based process | 3.35E-05 | -3.3565856 |
| actin cytoskeleton organization | 3.20E-05 | -3.2071349 |
| cell-cell junction | 0.00138958 | -3 |
| regulation of cytoskeleton organization | 0.00027247 | -2.7136021 |
| actin filament organization | 0.00054807 | -2.3333333 |
| actin filament binding | 0.00621277 | -2.236068 |
| lamellipodium | 1.83E-07 | -4.5961941 |
| postsynaptic density | 2.90E-06 | -4.3817805 |
| Golgi vesicle transport | 1.82E-06 | -4.2426407 |
| mitochondrial matrix | 8.84E-05 | -4.1461399 |
| polymeric cytoskeletal fiber | 2.39E-05 | -4.0824829 |
| establishment of organelle localization | 2.67E-07 | -3.9617739 |
| actin cytoskeleton | 7.74E-06 | -3.6055513 |
| cell-substrate adherens junction | 7.78E-07 | -3.6055513 |
| membrane raft | 9.50E-05 | -3.6055513 |
| mitochondrial envelope | 9.59E-05 | -3.5300904 |
| protein localization to membrane | 6.37E-07 | -3.5 |
| cell leading edge | 4.02E-06 | -3.4641016 |
| melanosome | 3.47E-05 | -3.4641016 |
| monocarboxylic acid metabolic process | 0.00040434 | -3.4641016 |
| cytoplasmic vesicle membrane | 0.00516202 | -3.3166248 |
| ficolin-1-rich granule | 0.00010057 | -3.3166248 |
| microtubule cytoskeleton organization | 0.00043204 | -3.3166248 |
| regulated exocytosis | 0.00633188 | -3.3166248 |
| vesicle membrane | 0.00627453 | -3.3166248 |
| regulation of GTPase activity | 0.00046749 | -3.2071349 |
| actin binding | 0.00021071 | -3.1622777 |
| cell body | 0.00161024 | -3.1622777 |
| axon | 5.06E-05 | -3.0508511 |
| supramolecular fiber organization | 0.00014419 | -3.0508511 |
| dendrite | 0.00025348 | -3 |
| ER to Golgi vesicle-mediated transport | 5.00E-06 | -3 |
| localization within membrane | 0.00021982 | -3 |
| membrane microdomain | 9.73E-05 | -3 |
| membrane region | 0.0001287 | -3 |
| neuronal cell body | 0.00238305 | -3 |
| organelle localization | 3.22E-06 | -3 |
| small GTPase mediated signal transduction | 0.00595031 | -3 |
| cytoplasmic region | 3.77E-05 | -2.8867513 |
| positive regulation of hydrolase activity | 0.00169731 | -2.8867513 |
| axon part | 0.00169745 | -2.8284271 |
| cell cortex | 0.00042414 | -2.8284271 |
| cell growth | 0.00730375 | -2.8284271 |
| coated vesicle | 0.00028394 | -2.8284271 |
| granulocyte activation | 0.00888403 | -2.8284271 |
| neutrophil activation | 0.00839491 | -2.8284271 |
| neutrophil activation involved in immune response | 0.00747772 | -2.8284271 |
| neutrophil degranulation | 0.00721793 | -2.8284271 |
| neutrophil mediated immunity | 0.00849108 | -2.8284271 |
| positive regulation of GTPase activity | 0.0024386 | -2.8284271 |
| Ras protein signal transduction | 0.00454474 | -2.8284271 |
| regulation of lipid metabolic process | 0.00262904 | -2.8284271 |
| ruffle | 7.47E-06 | -2.8284271 |
| site of polarized growth | 8.47E-06 | -2.8284271 |
| regulation of actin filament-based process | 1.63E-05 | -2.7136021 |
| cell projection assembly | 1.05E-05 | -2.6726124 |
| plasma membrane bounded cell projection assembly | 8.38E-06 | -2.6726124 |
| postsynapse | 1.26E-05 | -2.6726124 |
| actomyosin structure organization | 0.00016304 | -2.6457513 |
| carboxylic acid catabolic process | 0.00111634 | -2.6457513 |
| cell cortex part | 9.72E-05 | -2.6457513 |
| cell junction organization | 0.00151223 | -2.6457513 |
| distal axon | 0.00134254 | -2.6457513 |
| fatty acid metabolic process | 0.00701602 | -2.6457513 |
| growth cone | 6.14E-05 | -2.6457513 |
| microtubule associated complex | 0.00025387 | -2.6457513 |
| organic acid catabolic process | 0.00111634 | -2.6457513 |
| viral life cycle | 0.0030098 | -2.6457513 |
| cellular amino acid metabolic process | 5.45E-05 | -2.5298221 |
| regulation of cell morphogenesis | 0.00050675 | -2.5298221 |
| cell junction assembly | 0.00276342 | -2.4494897 |
| coated vesicle membrane | 0.00066108 | -2.4494897 |
| establishment or maintenance of cell polarity | 0.00145283 | -2.4494897 |
| pigment granule | 3.47E-05 | -2.4494897 |
| positive regulation of cell morphogenesis involved in differentiation | 0.00027238 | -2.4494897 |
| regulation of lipid biosynthetic process | 0.00102406 | -2.4494897 |
| axon development | 0.00298 | -2.3333333 |
| axonogenesis | 0.0015873 | -2.3333333 |
| regulation of actin cytoskeleton organization | 0.00017563 | -2.3333333 |
| regulation of supramolecular fiber organization | 0.00020833 | -2.3333333 |
| dendritic tree | 0.000261 | -2.3094011 |
| adherens junction organization | 0.00140018 | -2.236068 |
| alpha-amino acid metabolic process | 0.00950052 | -2.236068 |
| axon extension | 0.00068381 | -2.236068 |
| cortical actin cytoskeleton | 0.00017427 | -2.236068 |
| cortical cytoskeleton | 0.00056449 | -2.236068 |
| endoplasmic reticulum-Golgi intermediate compartment | 0.00082107 | -2.236068 |
| ficolin-1-rich granule lumen | 0.00076391 | -2.236068 |
| interaction with host | 0.00744106 | -2.236068 |
| monocarboxylic acid catabolic process | 0.00101178 | -2.236068 |
| motor activity | 0.00115601 | -2.236068 |
| neuron projection extension | 0.00291498 | -2.236068 |
| oxidoreductase activity, acting on CH-OH group of donors | 0.00091272 | -2.236068 |
| oxidoreductase activity, acting on the CH-OH group of donors, NAD or NADP as acceptor | 0.00065856 | -2.236068 |
| regulation of protein polymerization | 0.00866702 | -2.236068 |
| regulation of steroid biosynthetic process | 0.00022454 | -2.236068 |
| regulation of steroid metabolic process | 0.00076391 | -2.236068 |
| spindle organization | 0.00459678 | -2.236068 |
| steroid biosynthetic process | 0.00559591 | -2.236068 |
| cell morphogenesis involved in differentiation | 0.00010441 | -2.1380899 |
| protein domain specific binding | 6.09E-05 | -2.1380899 |
| cofactor binding | 0.00949882 | -2.1213203 |
| protein localization to cell periphery | 0.00047174 | -2.1213203 |
| protein-containing complex localization | 0.00022964 | -2.1213203 |
| regulation of actin filament organization | 0.00013893 | -2.1213203 |
| tubulin binding | 0.00077924 | -2.1213203 |
| asymmetric synapse | 3.26E-06 | -2.1105794 |
| microtubule | 3.01E-05 | -2.1105794 |
| neuron to neuron synapse | 6.05E-06 | -2.1105794 |
| nucleoside phosphate metabolic process | 0.00294356 | -2.1105794 |
| nucleotide metabolic process | 0.00273151 | -2.1105794 |
| postsynaptic specialization | 5.73E-06 | -2.1105794 |
| purine nucleotide metabolic process | 0.00051484 | -2.1105794 |
| purine-containing compound metabolic process | 0.00085819 | -2.1105794 |
| regulation of cell projection organization | 0.00267325 | -2.1105794 |
| regulation of plasma membrane bounded cell projection organization | 0.00239686 | -2.1105794 |

**Table S6: Significantly overrepresented GO (Gene Ontology) terms in the anterior cingulate cortex (ACC) of bipolar disorder (BD) patients**

| **GO term** | **adj_pval** | **z-score** |
| --- | --- | --- |
| actin cytoskeleton organization | 0.00479397 | 2.12132034 |
| mRNA processing | 0.00551581 | 2.64575131 |
| mRNA splicing, via spliceosome | 0.00074951 | 2.64575131 |
| RNA splicing | 0.00253222 | 2.64575131 |
| RNA splicing, via transesterification reactions | 0.00078489 | 2.64575131 |
| RNA splicing, via transesterification reactions with bulged adenosine as nucleophile | 0.00074951 | 2.64575131 |

**Table S7: Major functions of key proteins observed with significant changes in proteomics analysis of the dorsolateral prefrontal cortex (DLPFC) and anterior cingulate cortex (ACC) of mood disorder patients**

| **Protein name** | **Protein function** | **References** | **Medication effect** |
| --- | --- | --- | --- |
| COX5B | Chronic stress is one of the important risk factors for depression. Increased COX5B was reported to be part of the stress-protection mechanism within the hippocampal CA region in a rat model of depression. | (4, 5) | - no data |
| NDUFA10 | Is the accessory and non-catalytic subunit of the mitochondrial membrane respiratory chain NADH dehydrogenase (Complex I), which plays a role in the transfer of electrons from NADH to the respiratory chain. | (6) | - no data |
| PFKP | It (1) catalyzes the phosphorylation of D-fructose 6-phosphate to fructose 1,6-bisphosphate by ATP and (2) regulates glucose metabolism. | (7) | ↑ |
| OGDH | 2-oxoglutarate dehydrogenase is a component of the 2-oxoglutarate dehydrogenase complex, which catalyzes the overall conversion of 2-oxoglutarate to succinyl-CoA and CO_2_. The 2-oxoglutarate dehydrogenase complex is mainly active in the mitochondrion. | (8, 9) | - no data |
| SDHA | Flavoprotein subunit of succinate dehydrogenase (SDH) that is involved in complex II of the mitochondrial electron transport chain and is responsible for transferring electrons from succinate to ubiquinone (coenzyme Q). | (10) | - no data |
| CNTN2 | Contributes to the organization of axonal domains at nodes of Ranvier by maintaining voltage-gated potassium channels at the juxtaparanodal region when in conjunction with another transmembrane protein, CNTNAP2. | (11) | - no data |
| HSP90AA1 | It (1) participates in axon extension and cytokine-mediated signal pathways and (2) binds bacterial lipopolysaccharide (LPS) and mediates LPS-induced inflammatory response, including TNF secretion by monocytes. | (12) | - no data |
| GDI2, COPB1 and STOM | Are related to neutrophil degranulation. |  | - no data |
| MSN | When (1) activated with phosphorylation of C-terminal threonine, leads to interaction between MSN and F-actin, thus enhancing cytoskeletal rearrangement, which regulates cellular processes including cell shape determination, membrane transport, and signal transduction and (2) participates in immunologic synapse formation. | (13-16) | - no data |
| PFN1 | Binds to actin and affects the structure of the cytoskeleton. | (17) | ↑ |
| PDCD6IP | Binds F-actin and thus regulates actin cytoskeleton assembly. | (18) | - no data |
| DBN1 | Is an actin cytoskeleton-organizing protein contributing to the formation of cell projections | (19) | - no data |
| MYH9 | Contributes to cytoskeleton reorganization and focal contact formation during cell spreading. | (20) | - no data |
| DBNL | Participates in the reorganization of the actin cytoskeleton, in formation of cell projections such as dendrites, and in synapse formation via its interaction with protein cordon-bleu (COBL), which plays an important role in the reorganization of the actin cytoskeleton. | (21) | - no data |
| SYN3 | Involved in the regulation of neurotransmitter release and synaptogenesis | (22) |  |
| PDLIM5 | Is present in the postsynaptic density, where it promotes decreased dendritic spine head size and longer, filopodia-like morphology | (23) |  |
| PRNP | Plays an important role in neuronal development and synaptic plasticity | (24) |  |
| STXBP1 | Participates in the regulation of synaptic vesicle docking and fusion through interaction with GTP-binding proteins | (25) |  |
| GOLGA2 | Acts as a vesicle tether that facilitates vesicle fusion to the Golgi membrane | (26) |  |
| VPS4A | The endosomal sorting complexes required for transport-III (ESCRT-III) proteins bind directly to VPS4 proteins and recruit the VPS4 AAA ATPases to sites of multivesicular body (MVB) vesicle formation | (27, 28) |  |

**Table S8: Significantly overrepresented GO (Gene Ontology) terms in the dorsolateral prefrontal cortex (DLPFC) and anterior cingulate cortex of major depressive disorder (MDD) patients compared with bipolar disorder (BD) patients**

| **GO term** | **adj_pval** | **z-score** |
| --- | --- | --- |
| **Anterior cingulate cortex (ACC)** |  |  |
| Golgi vesicle transport | 9.14E-08 | -4.26401 |
| regulation of vesicle-mediated transport | 1.30E-08 | -4.12311 |
| regulation of secretion | 9.24E-08 | -3.63803 |
| axo-dendritic transport | 3.82E-06 | -3.53553 |
| regulation of secretion by cell | 1.35E-07 | -3.5 |
| secretory granule lumen | 6.83E-10 | -3.35659 |
| cytoplasmic vesicle lumen | 7.75E-10 | -3.35659 |
| vesicle lumen | 8.44E-10 | -3.35659 |
| protein kinase binding | 8.70E-06 | -3.35659 |
| cell leading edge | 1.46E-07 | -3.33333 |
| Golgi membrane | 0.001318 | -3.31662 |
| intracellular protein transport | 6.05E-08 | -3.26599 |
| regulation of exocytosis | 3.26E-07 | -3.16228 |
| side of membrane | 0.005485 | -3.16228 |
| glutamatergic synapse | 4.39E-08 | -3.05085 |
| fatty acid metabolic process | 5.87E-08 | -3.05085 |
| blood coagulation | 7.45E-07 | -3 |
| coagulation | 8.20E-07 | -3 |
| hemostasis | 9.46E-07 | -3 |
| wound healing | 0.000122 | -3 |
| regulation of body fluid levels | 0.000289 | -3 |
| regulation of cellular localization | 1.43E-05 | -3 |
| positive regulation of secretion | 1.93E-07 | -2.88675 |
| vesicle localization | 3.45E-06 | -2.85774 |
| regulation of protein transport | 2.06E-07 | -2.84019 |
| monocarboxylic acid metabolic process | 1.85E-07 | -2.84019 |
| platelet activation | 8.88E-08 | -2.82843 |
| response to calcium ion | 1.99E-06 | -2.82843 |
| kinase binding | 4.03E-07 | -2.82843 |
| primary lysosome | 3.45E-06 | -2.82843 |
| azurophil granule | 3.45E-06 | -2.82843 |
| vacuolar lumen | 8.10E-06 | -2.82843 |
| vacuolar membrane | 0.004658 | -2.82843 |
| positive regulation of secretion by cell | 6.09E-07 | -2.7136 |
| positive regulation of protein transport | 1.17E-06 | -2.7136 |
| positive regulation of establishment of protein localization | 1.97E-06 | -2.7136 |
| response to wounding | 4.31E-05 | -2.7136 |
| extracellular matrix | 0.000482 | -2.7136 |
| external encapsulating structure | 0.000489 | -2.7136 |
| regulation of MAPK cascade | 0.002187 | -2.7136 |
| endomembrane system organization | 0.000175 | -2.7136 |
| response to inorganic substance | 1.82E-06 | -2.67261 |
| platelet aggregation | 5.04E-09 | -2.64575 |
| homotypic cell-cell adhesion | 4.37E-08 | -2.64575 |
| positive regulation of exocytosis | 5.35E-07 | -2.64575 |
| endoplasmic reticulum to Golgi vesicle-mediated transport | 6.77E-06 | -2.64575 |
| vesicle organization | 0.001217 | -2.64575 |
| circulatory system process | 0.0006 | -2.52982 |
| carbohydrate metabolic process | 0.000368 | -2.52982 |
| cell cortex | 1.37E-05 | -2.52982 |
| glycogen catabolic process | 1.31E-07 | -2.52357 |
| regulation of establishment of protein localization | 6.45E-08 | -2.5 |
| axon | 7.87E-07 | -2.5 |
| membrane organization | 1.14E-05 | -2.5 |
| response to metal ion | 1.43E-07 | -2.49615 |
| positive regulation of protein localization | 2.10E-06 | -2.49615 |
| regulation of wound healing | 0.000125 | -2.44949 |
| blood microparticle | 0.000216 | -2.44949 |
| regulation of blood circulation | 0.00378 | -2.44949 |
| vascular process in circulatory system | 0.004308 | -2.44949 |
| protein localization to cell periphery | 0.002379 | -2.44949 |
| Golgi organization | 0.000209 | -2.44949 |
| regulation of regulated secretory pathway | 0.000166 | -2.44949 |
| regulation of protein secretion | 1.68E-05 | -2.33333 |
| blood circulation | 0.000549 | -2.33333 |
| cell-cell adhesion | 0.003895 | -2.33333 |
| secretory granule membrane | 8.78E-05 | -2.33333 |
| negative regulation of extrinsic apoptotic signaling pathway | 0.000251 | -2.23607 |
| cell-matrix adhesion | 0.001026 | -2.23607 |
| regulation of tube diameter | 0.001376 | -2.23607 |
| blood vessel diameter maintenance | 0.001376 | -2.23607 |
| regulation of tube size | 0.00142 | -2.23607 |
| regulation of extrinsic apoptotic signaling pathway | 0.001971 | -2.23607 |
| extracellular matrix structural constituent | 0.003267 | -2.23607 |
| cell-substrate adhesion | 0.004761 | -2.23607 |
| protein localization to plasma membrane | 0.005546 | -2.23607 |
| post-Golgi vesicle-mediated transport | 0.00024 | -2.23607 |
| endoplasmic reticulum-Golgi intermediate compartment | 0.000992 | -2.23607 |
| regulation of neurotransmitter transport | 0.000276 | -2.23607 |
| lysosomal lumen | 0.000251 | -2.23607 |
| vesicle budding from membrane | 2.14E-05 | -2.23607 |
| regulation of cell-substrate adhesion | 3.97E-05 | -2.12132 |
| regulation of hormone secretion | 6.17E-05 | -2.12132 |
| regulation of ERK1 and ERK2 cascade | 0.000467 | -2.12132 |
| regulation of apoptotic signaling pathway | 0.001358 | -2.12132 |
| collagen-containing extracellular matrix | 0.003404 | -2.12132 |
| positive regulation of cell adhesion | 0.004477 | -2.12132 |
| positive regulation of MAPK cascade | 0.007741 | -2.12132 |
| regulation of hormone levels | 0.000152 | -2.11058 |
| **Dorsolateral prefrontal cortex (DLPFC)** |  |  |
| generation of precursor metabolites and energy | 7.51E-06 | 2.713602 |
| type 5 metabotropic glutamate receptor binding | 1.90E-09 | 2.32379 |
| neuron to neuron synapse | 2.53E-09 | 2.309401 |
| myofibril | 0.001126 | 2.236068 |
| contractile fiber | 0.001361 | 2.236068 |
| muscle cell differentiation | 0.00197 | 2.236068 |
| postsynaptic density | 1.21E-08 | 2.110579 |
| asymmetric synapse | 1.42E-08 | 2.110579 |
| postsynaptic specialization | 2.55E-08 | 2.110579 |
| modulation of chemical synaptic transmission | 2.52E-07 | 2.110579 |
| regulation of trans-synaptic signaling | 2.58E-07 | 2.110579 |

**
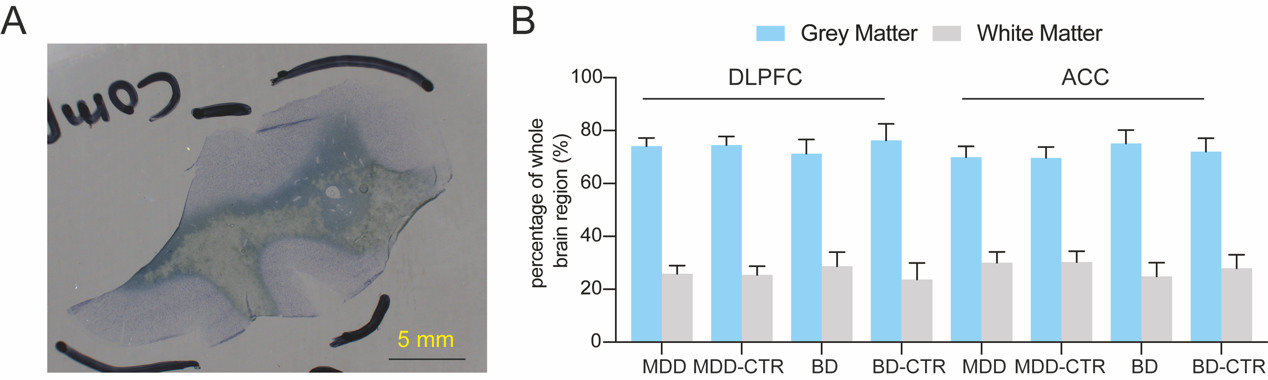
**

**Supplementary Figure 1:** Quantification of the percentages of grey matter and white matter in the dorsolateral prefrontal cortex (DLPFC) and anterior cingulate cortex (ACC) of major depressive disorder (MDD) and bipolar disorder (BD) patients. (A-B) The percentage of grey matter and white matter was monitored by computerized image system. The ratios of grey matter/white matter were around 2·7 both in the DLPFC and the ACC of MDD, BD and their respective controls, without significant differences. Data in B were shown as mean ± SEM.

**
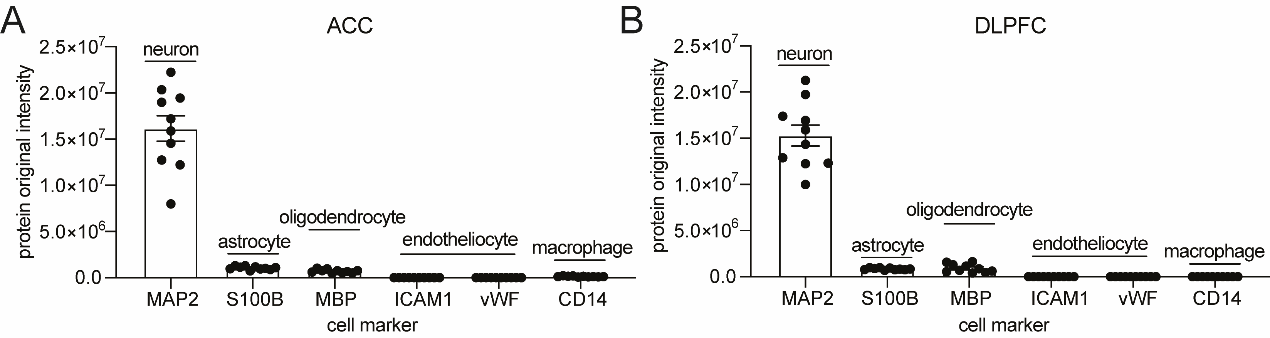
**

**Supplementary Figure 2:** Cell type determination in the dorsolateral prefrontal cortex (DLPFC) and anterior cingulate cortex (ACC) of major depressive disorder (MDD) and bipolar disorder (BD) patients. (A-B) Protein expression level of specific markers of different cell types in the DLPFC and ACC (One-way ANOVA: DLPFC, p < 0.0001; ACC, p < 0·0001). Note that the neuronal protein, i.e. MAP2, has the most significant enrichment. Data were shown as mean ± SEM.

**
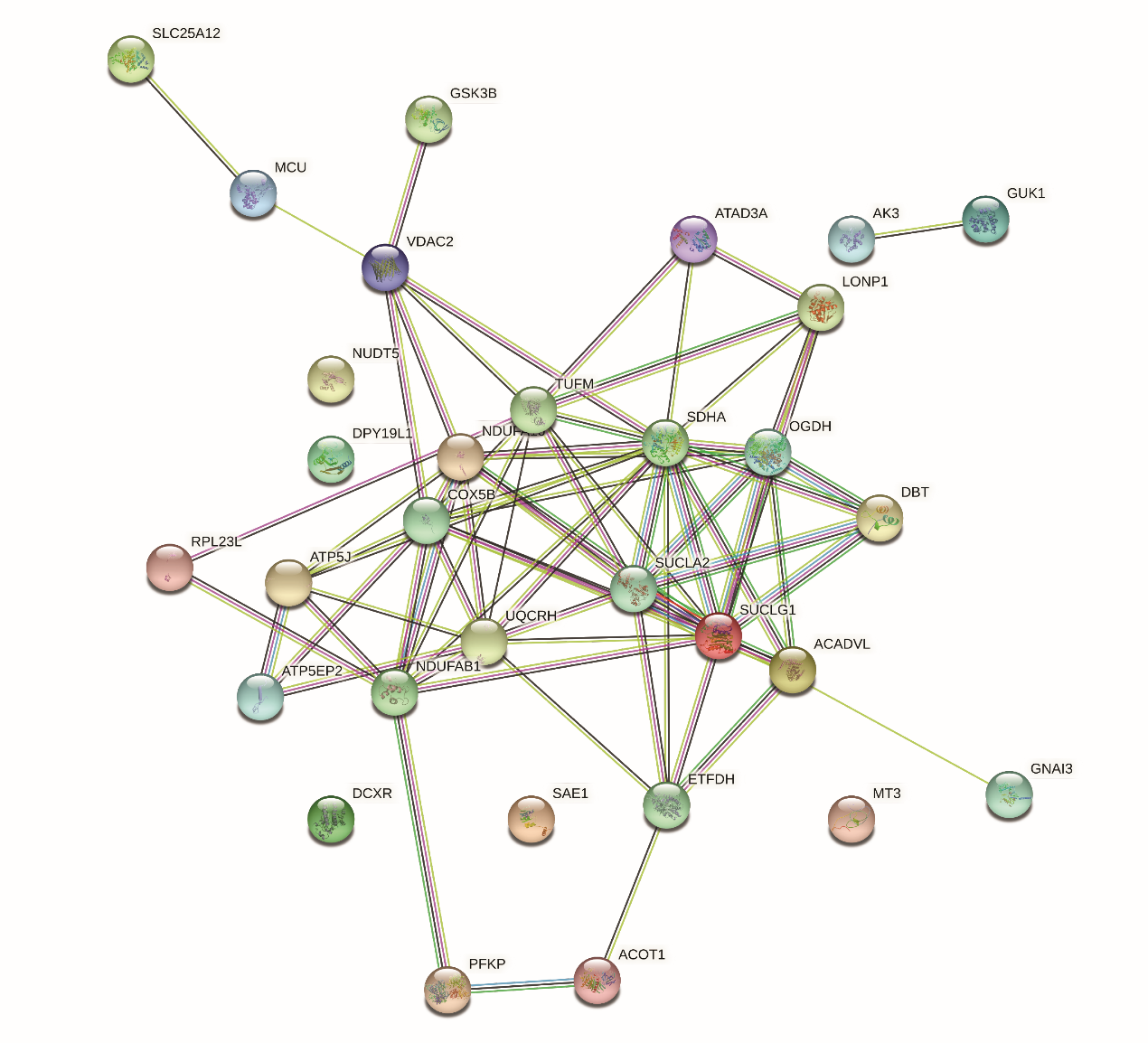
**

**Supplementary Figure 3:** Interaction network of differentially expressed proteins related to energy metabolism in the DLPFC of MDD patients. Balls represent proteins, and lines represent interactions between proteins. Protein-protein interactions for the differentially expressed proteins identified by TMT-based proteomics were analyzed with the STRING database.


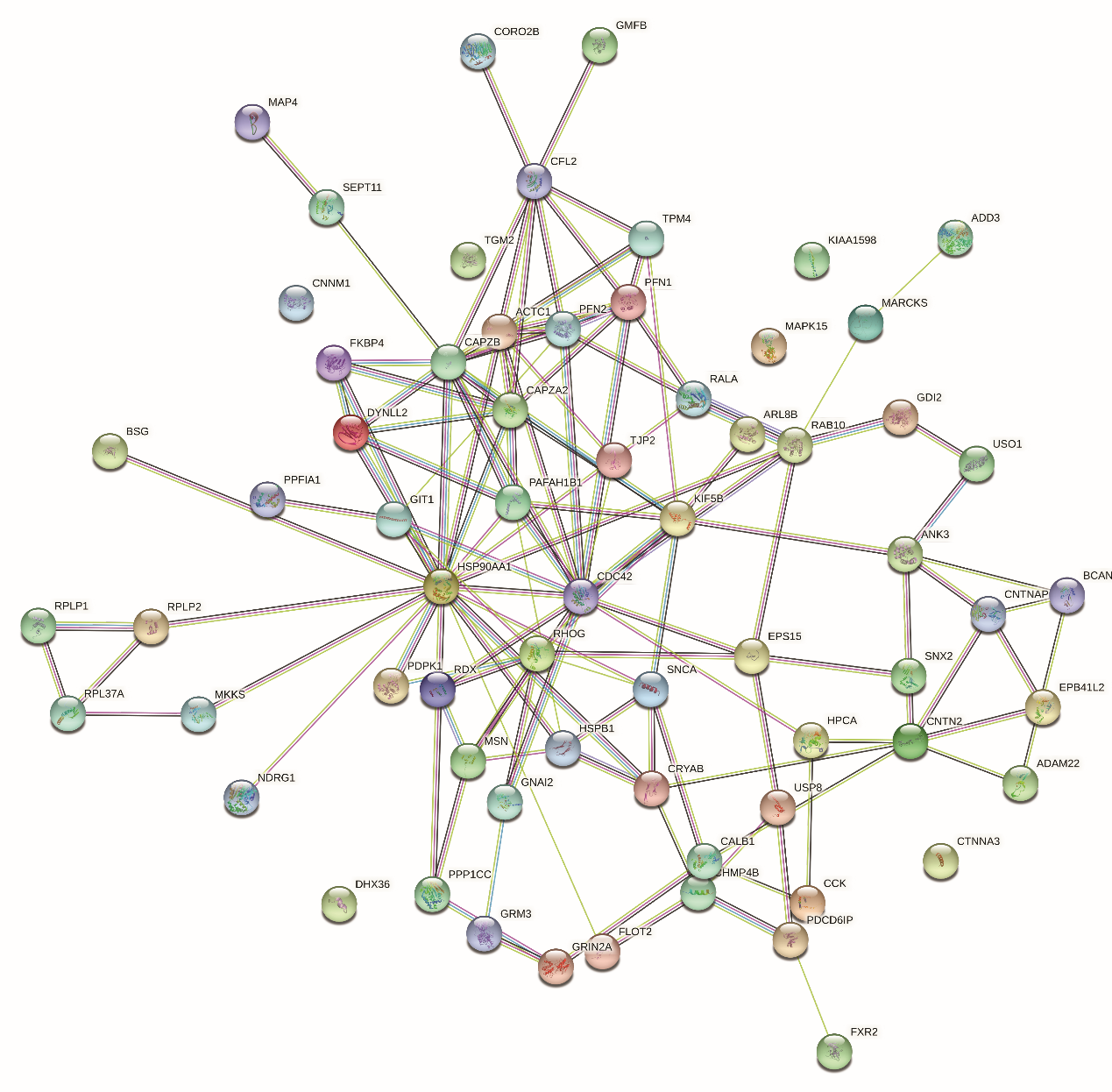


**Supplementary Figure 4:** Interaction network of differentially expressed proteins related to tissue remodeling, immune response and synaptic function in the ACC of MDD patients. Balls represent proteins, and lines represent interactions between proteins. Protein-protein interactions for the differentially expressed proteins identified by TMT-based proteomics were analyzed with the STRING database.


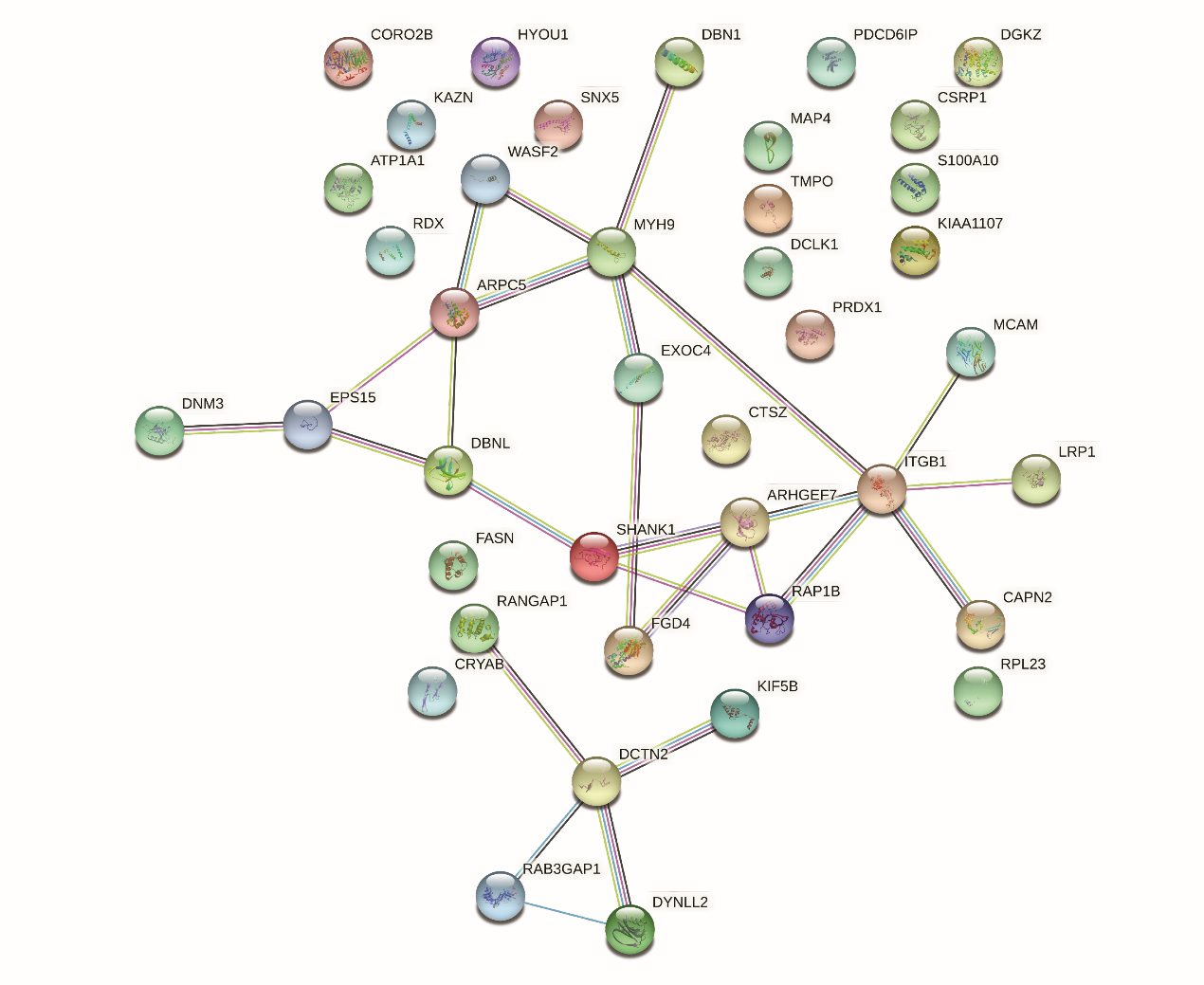


**Supplementary Figure 5:** Interaction network of differentially expressed proteins related to tissue remodeling and neuronal projection in the DLPFC of BD patients. Balls represent proteins, and lines represent interactions between proteins. Protein-protein interactions for the differentially expressed proteins identified by TMT-based proteomics were analyzed with the STRING database.

**
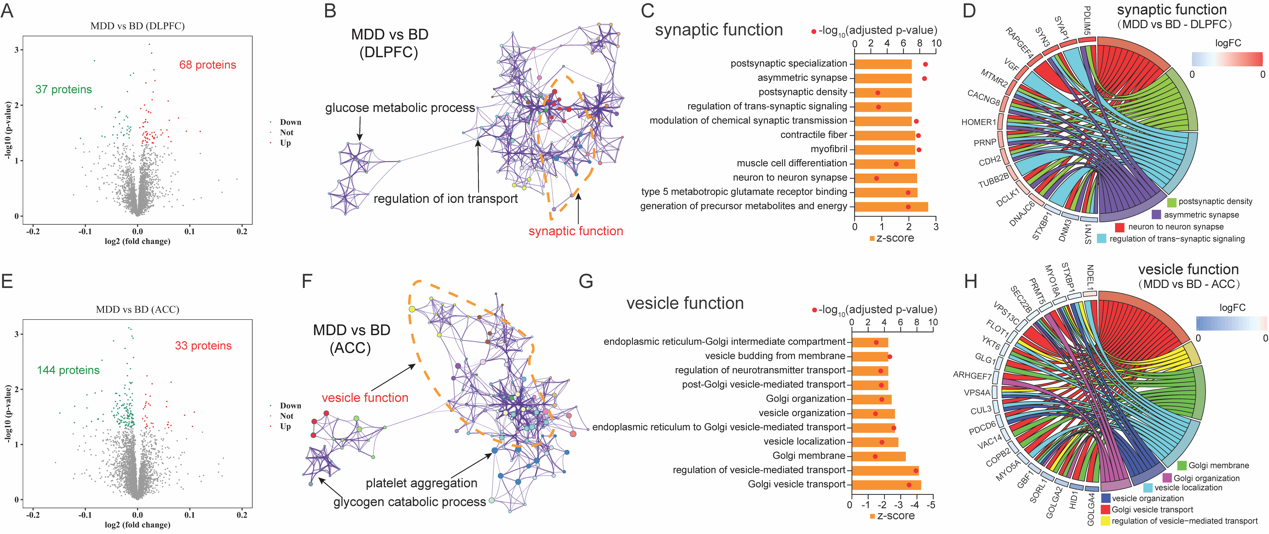
**

**Supplementary Figure 6:** Distinct functional cluster alterations in the dorsolateral prefrontal cortex (DLPFC) and anterior cingulate cortex (ACC) of major depressive disorder (MDD) patients compared with bipolar disorder (BD) patients. (A) Differential expression analysis was performed on proteomics data. Volcano plot shows the differentially expressed proteins (DEPs; adjusted p-value < 0·05) in the DLPFC of MDD patients compared with BD patients. 105 DEPs in the DLPFC were identified, among which 37 proteins were downregulated and 68 proteins were upregulated. (B) Gene ontology (GO) enrichment analysis was performed using Metascape on the 105 DEPs in the DLPFC of MDD patients. The significantly overrepresented (adjusted p-value < 0·01) GO terms were grouped into color-coded clusters based on their membership similarities and rendered as a network plot. Each node represents an enriched term, and one representative term is shown for each cluster. Terms with a similarity > 0·3 are connected by edges. (C) The activation status of biological processes related to synaptic function was assessed by calculating their activation z-scores using GOplot. The 11 GO terms are shown associated with synaptic function that were predicted to be strongly activated (z-score > 2) in the DLPFC of MDD patients compared with BD patients. (D) Proteins related to synaptic function were explored for their involvement in 4 functional sub-categories. Shown are proteins associated with synaptic functional sub-categories, displayed as a Circos plot. (E) Volcano plot shows the DEPs in the ACC of MDD patients compared with BD patients. 177 DEPs in the ACC were identified, among which 144 proteins were downregulated and 33 proteins were upregulated. (F) GO enrichment analysis shows the most involved functional clusters is vesicle function in the ACC of MDD patients compared with BD patients. (G) The suppression status of biological processes related to vesicle function was assessed by calculating their suppression z-scores using GOplot. The 11 GO terms associated with vesicle function were predicted to be strongly decreased (z-score < -2) in ACC of MDD patients. (H) Proteins related to vesicle function were explored for their involvement in 6 functional sub-categories. Shown are proteins associated with vesicle functional sub-categories, displayed as a Circos plot.

**Reference:**

1. Wessel D, Flugge UI. A method for the quantitative recovery of protein in dilute solution in the presence of detergents and lipids. Anal Biochem. 1984;138(1):141-3.

2. Horton AC, Racz B, Monson EE, Lin AL, Weinberg RJ, Ehlers MD. Polarized secretory trafficking directs cargo for asymmetric dendrite growth and morphogenesis. Neuron. 2005;48(5):757-71.

3. Jan YN, Jan LY. Branching out: mechanisms of dendritic arborization. Nat Rev Neurosci. 2010;11(5):316-28.

4. Henningsen K, Palmfeldt J, Christiansen S, Baiges I, Bak S, Jensen ON, et al. Candidate hippocampal biomarkers of susceptibility and resilience to stress in a rat model of depression. Mol Cell Proteomics. 2012;11(7):M111 016428.

5. Pittenger C, Duman RS. Stress, depression, and neuroplasticity: a convergence of mechanisms. Neuropsychopharmacology. 2008;33(1):88-109.

6. Stroud DA, Surgenor EE, Formosa LE, Reljic B, Frazier AE, Dibley MG, et al. Accessory subunits are integral for assembly and function of human mitochondrial complex I. Nature. 2016;538(7623):123-6.

7. Shen J, Jin Z, Lv H, Jin K, Jonas K, Zhu C, et al. PFKP is highly expressed in lung cancer and regulates glucose metabolism. Cell Oncol (Dordr). 2020;43(4):617-29.

8. Armstrong CT, Anderson JL, Denton RM. Studies on the regulation of the human E1 subunit of the 2-oxoglutarate dehydrogenase complex, including the identification of a novel calcium-binding site. Biochem J. 2014;459(2):369-81.

9. Wang Y, Guo YR, Liu K, Yin Z, Liu R, Xia Y, et al. KAT2A coupled with the alpha-KGDH complex acts as a histone H3 succinyltransferase. Nature. 2017;552(7684):273-7.

10. Renkema GH, Wortmann SB, Smeets RJ, Venselaar H, Antoine M, Visser G, et al. SDHA mutations causing a multisystem mitochondrial disease: novel mutations and genetic overlap with hereditary tumors. Eur J Hum Genet. 2015;23(2):202-9.

11. Stogmann E, Reinthaler E, Eltawil S, El Etribi MA, Hemeda M, El Nahhas N, et al. Autosomal recessive cortical myoclonic tremor and epilepsy: association with a mutation in the potassium channel associated gene CNTN2. Brain. 2013;136(Pt 4):1155-60.

12. Triantafilou K, Triantafilou M, Dedrick RL. A CD14-independent LPS receptor cluster. Nat Immunol. 2001;2(4):338-45.

13. Huang L, Wong TY, Lin RC, Furthmayr H. Replacement of threonine 558, a critical site of phosphorylation of moesin in vivo, with aspartate activates F-actin binding of moesin. Regulation by conformational change. J Biol Chem. 1999;274(18):12803-10.

14. Iontcheva I, Amar S, Zawawi KH, Kantarci A, Van Dyke TE. Role for moesin in lipopolysaccharide-stimulated signal transduction. Infect Immun. 2004;72(4):2312-20.

15. Lagresle-Peyrou C, Luce S, Ouchani F, Soheili TS, Sadek H, Chouteau M, et al. X-linked primary immunodeficiency associated with hemizygous mutations in the moesin (MSN) gene. J Allergy Clin Immunol. 2016;138(6):1681-9 e8.

16. Urzainqui A, Serrador JM, Viedma F, Yanez-Mo M, Rodriguez A, Corbi AL, et al. ITAM-based interaction of ERM proteins with Syk mediates signaling by the leukocyte adhesion receptor PSGL-1. Immunity. 2002;17(4):401-12.

17. Shao J, Welch WJ, Diprospero NA, Diamond MI. Phosphorylation of profilin by ROCK1 regulates polyglutamine aggregation. Mol Cell Biol. 2008;28(17):5196-208.

18. Pan S, Wang R, Zhou X, He G, Koomen J, Kobayashi R, et al. Involvement of the conserved adaptor protein Alix in actin cytoskeleton assembly. J Biol Chem. 2006;281(45):34640-50.

19. Perez-Martinez M, Gordon-Alonso M, Cabrero JR, Barrero-Villar M, Rey M, Mittelbrunn M, et al. F-actin-binding protein drebrin regulates CXCR4 recruitment to the immune synapse. J Cell Sci. 2010;123(Pt 7):1160-70.

20. Betapudi V. Myosin II motor proteins with different functions determine the fate of lamellipodia extension during cell spreading. PLoS One. 2010;5(1):e8560.

21. Inoue S, Hayashi K, Fujita K, Tagawa K, Okazawa H, Kubo KI, et al. Drebrin-like (Dbnl) Controls Neuronal Migration via Regulating N-Cadherin Expression in the Developing Cerebral Cortex. J Neurosci. 2019;39(4):678-91.

22. Longhena F, Faustini G, Brembati V, Pizzi M, Benfenati F, Bellucci A. An updated reappraisal of synapsins: structure, function and role in neurological and psychiatric disorders. Neurosci Biobehav Rev. 2021;130:33-60.

23. Herrick S, Evers DM, Lee JY, Udagawa N, Pak DT. Postsynaptic PDLIM5/Enigma Homolog binds SPAR and causes dendritic spine shrinkage. Mol Cell Neurosci. 2010;43(2):188-200.

24. Caiati MD, Safiulina VF, Fattorini G, Sivakumaran S, Legname G, Cherubini E. PrPC controls via protein kinase A the direction of synaptic plasticity in the immature hippocampus. J Neurosci. 2013;33(7):2973-83.

25. Swanson DA, Steel JM, Valle D. Identification and characterization of the human ortholog of rat STXBP1, a protein implicated in vesicle trafficking and neurotransmitter release. Genomics. 1998;48(3):373-6.

26. Kotecha U, Mistri M, Shah N, Shah PS, Gupta VA. Bi-allelic loss of function variants in GOLGA2 are associated with a complex neurological phenotype: Report of a second family. Clin Genet. 2021;100(6):748-51.

27. Babst M, Wendland B, Estepa EJ, Emr SD. The Vps4p AAA ATPase regulates membrane association of a Vps protein complex required for normal endosome function. EMBO J. 1998;17(11):2982-93.

28. Martin-Serrano J, Yarovoy A, Perez-Caballero D, Bieniasz PD. Divergent retroviral late-budding domains recruit vacuolar protein sorting factors by using alternative adaptor proteins. Proc Natl Acad Sci U S A. 2003;100(21):12414-9.
